# Saccharobisindole, Neoasterric Methyl Ester, and 7-Chloro-4(1H)-quinolone: Three New Compounds Isolated from the Marine Bacterium *Saccharomonospora* sp.

**DOI:** 10.3390/md20010035

**Published:** 2021-12-29

**Authors:** Sohee Kim, Tu Cam Le, Sang-Ah Han, Prima F. Hillman, Ahreum Hong, Sunghoon Hwang, Young Eun Du, Hiyoung Kim, Dong-Chan Oh, Sun-Shin Cha, Jihye Lee, Sang-Jip Nam, William Fenical

**Affiliations:** 1 The Graduate School of Industrial Pharmaceutical Sciences, Ewha Womans University, Seoul 03760, Korea; chi0736@naver.com (S.K.); tkddk9559@naver.com (S.-A.H.); lyzenne@naver.com (A.H.); 2 Laboratory of Advanced Materials Chemistry, Advanced Institute of Materials Science, Ton Duc Thang University, Ho Chi Minh City 700000, Vietnam; lecamtu@tdtu.edu.vn; 3 Faculty of Applied Sciences, Ton Duc Thang University, Ho Chi Minh City 700000, Vietnam; 4 Department of Chemistry and Nanoscience, Ewha Womans University, Seoul 03760, Korea; primafitriah@gmail.com (P.F.H.); cha-jung@ewha.ac.kr (S.-S.C.); 5 Laboratories of Marine New Drugs, REDONE Seoul, Seoul 08594, Korea; 6 Natural Products Research Institute, College of Pharmacy, Seoul National University, Seoul 08826, Korea; sunghooi@snu.ac.kr (S.H.); dye0302@snu.ac.kr (Y.E.D.); dongchanoh@snu.ac.kr (D.-C.O.); 7 Department of Biomedical Science and Engineering, Konkuk University, Seoul 05029, Korea; reihyoung@konkuk.ac.kr; 8 Center for Marine Biotechnology and Biomedicine, Scripps Institution of Oceanography, University of California-San Diego, La Jolla, CA 92093-0204, USA

**Keywords:** *Saccharomonospora* sp., marine natural products, antibacterial activity

## Abstract

Analysis of the chemical components from the culture broth of the marine bacterium *Saccharomonospora* sp. CNQ-490 has yielded three novel compounds: saccharobisindole (**1**), neoasterric methyl ester (**2**), and 7-chloro-4(*1H*)-quinolone (**3**), in addition to acremonidine E (**4**), pinselin (**5**), penicitrinon A (**6**), and penicitrinon E (**7**). The chemical structures of the three novel compounds were elucidated by the interpretation of 1D, 2D nuclear magnetic resonance (NMR), and high-resolution mass spectrometry (HRMS) data. Compound **2** generated weak inhibition activity against *Bacillus subtilis* KCTC2441 and *Staphylococcus aureus* KCTC1927 at concentrations of 32 μg/mL and 64 μg/mL, respectively, whereas compounds **1** and **3** did not have any observable effects. In addition, compound **2** displayed weak anti-quorum sensing (QS) effects against *S. aureus* KCTC1927 and *Micrococcus luteus* SCO560.

## 1. Introduction

Actinomycetes, a group of aerobic and anaerobic Gram-positive mycelial bacteria, are known to produce a variety of bioactive secondary metabolites. In fact, more than 70% of the currently used antibiotics were originally isolated from *Streptomyces*, the largest genus of actinomycetes [1]. Rare actinomycetes have also been regarded as potential sources for the discovery of bioactive compounds, including antibiotics [1]. In the past five years, 31% of new bioactive compounds were isolated from rare actinomycete strains, although the *Streptomyces* genus continues to dominate this field, contributing 65% of the reported bioactive compounds [2]. 

**Figure 1:**
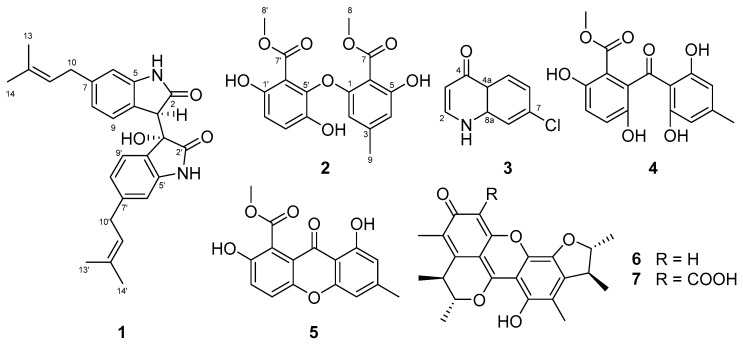
Chemical structures of saccharobisindole (**1**), neoasterric methyl ester (**2**), 7-chloro-4(*1H*)-quinolone (**3**), acremonidine E (**4**), pinselin (**5**), penicitrinon A (**6**), and penicitrinon E (**7**).

The genus *Saccharomonospora*, a rare actinomycete, was first described in 1971 [3,4] and only a few secondary metabolites have been isolated from this genus [5,6]. The strain CNQ-490, *Saccharomonospora* sp., was isolated from a sediment sample of La Jolla Submarine Canyon and previous chemical analyses of strain CNQ-490 led to the identification of seven novel secondary metabolites including lodopyridones A–C [7,8], sacchromonopyrones A–C [9], and saccharoquinoline [10]. Additionally, a genomic study of strain CNQ-490 demonstrated that this bacterium possessed a silent biosynthetic gene cluster, which led to the discovery of the novel antibiotic compounds taromycins A and B [11,12]. The identification of 19 biosynthetic gene clusters in this strain suggested that it could produce a wide structural diversity of secondary metabolites. Further characterization of the bioactive metabolites of strain CNQ-490 led to the discovery of saccharobisindole (**1**), neoasterric methyl ester (**2**), and 7-chloro-4(*1H*)-quinolone (**3**), in addition to four known natural products, including acremonidine E (**4**), pinselin (**5**), penicitrinon A (6), and penicitrinon E (**7**) (Figure 1). The present study elucidated the structure of compounds **1**–**3**, as well as the biological activities of **1**–**7**.

## 2. Results

Saccharobisindole (**1**) was obtained as a pale, yellow oil. The molecular formula of this compound (C_26_H_28_N_2_O_3_) was deduced from high-resolution fast atom bombardment mass spectrometry (HRFABMS) coupled with the detection of an ion at *m/z* 417.2181 [M+H]^+^ (calcd for C_26_H_29_N_2_O_3_, 417.2178) indicating 14 degrees of unsaturation. The infrared (IR) spectrum of this compound indicated the presence of a hydroxy group at 3431 cm^−1^, a carbonyl group at 1676 cm^−1^, and a double bond at 1638 cm^−1^. 

The ^1^H NMR spectrum of **1** exhibited two sets of 1,2,4-trisubstituted aromatic protons H-6 (*δ*_H_ 6.55, 1H, s), H-8 (*δ*_H_ 6.80, 1H, d, *J* = 7.7 Hz), H-9 (*δ*_H_ 7.35, 1H, d, *J* = 7.7 Hz), H-6′ (*δ*_H_ 6.53, 1H, s), H-8′ (*δ*_H_ 6.45, 1H, d, *J* = 7.6 Hz), and H-9′ (*δ*_H_ 6.09, 1H, d, *J* = 7.6 Hz), four methyl singlets H-13 (*δ*_H_ 1.72, 3H, s), H-14 (*δ*_H_ 1.69, 3H, s), H-13′ (*δ*_H_ 1.68, 3H, s), and H-14′ (*δ*_H_ 1.64, 3H, s), two triplet olefinic proton signals H-11 (*δ*_H_ 5.29, 1H, t, *J* = 7.3 Hz) and H-11′(*δ*_H_ 5.21, 1H, t, *J* = 7.5 Hz), two doublet protons H-10 (*δ*_H_ 3.30, 2H, d, *J* = 7.9 Hz) and H-10′ (*δ*_H_ 3.19, 2H, d, *J* = 7.5 Hz), and one singlet proton H-3 (*δ*_H_ 3.91, 1H, s). Furthermore, three exchangeable protons at NH-1 (*δ*_H_ 10.08), NH-1′ (*δ*_H_ 10.22), and 3′-OH (*δ*_H_ 6.40) were also observed in the ^1^H NMR spectrum. The analysis of ^13^C NMR and heteronuclear single quantum coherence (HSQC) spectroscopic data indicated two carbonyl C-2 (*δ*_C_ 174.6) and C-2′ (*δ*_C_ 177.3), eight sp^2^ quaternary C-4 (*δ*_C_ 123.2), C-5 (*δ*_C_ 143.4), C-7 (*δ*_C_ 141.8), C-12 (*δ*_C_ 131.8), C-4′ (*δ*_C_ 125.8), C-5′ (*δ*_C_ 142.9), C-7′ (*δ*_C_ 143.1), and C-12′ (*δ*_C_ 131.9), six aromatic methine C-6 (*δ*_C_ 108.8), C-8 (*δ*_C_ 120.9), C-9 (*δ*_C_ 126.2), C-6′ (*δ*_C_ 109.4), C-8′ (*δ*_C_ 120.8), and C-9′ (*δ*_C_ 123.5), two olefinic C-11 (*δ*_C_ 123.2) and C-11′ (*δ*_C_ 122.9), two methylene C-10 (*δ*_C_ 33.8) and C-10′ (*δ*_C_ 33.7), and four methyl singlet carbons C-13 (*δ*_C_ 25.5), C-14 (*δ*_C_ 17.7), C-13′ (*δ*_C_ 25.4), and C-14′ (*δ*_C_ 17.6), along with one sp^3^ methine group C-3 (*δ*_C_ 51.1) and one sp^3^ quaternary carbon C-3′ (*δ*_C_ 75.4) (Table 1).

Further interpretation of 2D NMR spectroscopic data including two-bond HMBC (heteronuclear multiple bond correlation) analysis allowed for the structural construction of **1**. Two sets of indolin-2-one moieties were identified by the interpretation of COSY (COrrelated SpectroscopY) and HMBC spectral data. The COSY cross peaks H-6/H-8/H-9 and H-6′/H-8′/H-9′ and the long-range HMBC correlations H-8 (*δ*_H_ 6.80)/ C-4 (*δ*_C_ 123.2), C-6 (*δ*_C_ 108.8); H-9 (*δ*_H_ 7.35)/ C-5 (*δ*_C_ 143.4), C-7 (*δ*_C_ 141.8); singlet aromatic proton H-6 (*δ*_H_ 6.55)/ C-4, C-8 (*δ*_C_ 120.9); H-8′ (*δ*_H_ 6.45)/ C-4′ (*δ*_C_ 125.8), C-6′ (*δ*_C_ 109.4); H-9′ (*δ*_H_ 6.09)/ C-5′ (*δ*_C_ 142.9), C-7′(*δ*_C_ 143.1); and H-6′ (*δ*_H_ 6.53)/ C-4′, C-8′(*δ*_C_ 120.8) confirmed the presence of two sets of 1,2,4-trisubstituted benzene moieties. Furthermore, two sets of 3-methylbut-2-enyl moieties were also identified from the interpretation of COSY and log-range HMBC analyses. COSY crosspeaks H-10/H-11 and H-10′/H-11′ and long-range HMBC correlations H-13 (*δ*_H_ 1.72) and H-14 (*δ*_H_ 1.69)/C-11 (*δ*_C_ 123.2), C-12 (*δ*_C_ 131.8); H-10 (*δ*_H_ 3.30)/ C-11 (*δ*_C_ 123.2), C-12 (*δ*_C_ 131.8); H-13′ (*δ*_H_ 1.68) and H-14′ (*δ*_H_ 1.64)/ C-11′ (*δ*_C_ 122.9), C-12′(*δ*_C_ 131.9); and H-10′ (*δ*_H_ 3.19)/ C-11′ (*δ*_C_ 122.9), C-12′ (*δ*_C_ 131.9) allowed for the assignment of two sets of 3-methylbut-2-enyl units.

**Figure 2:**
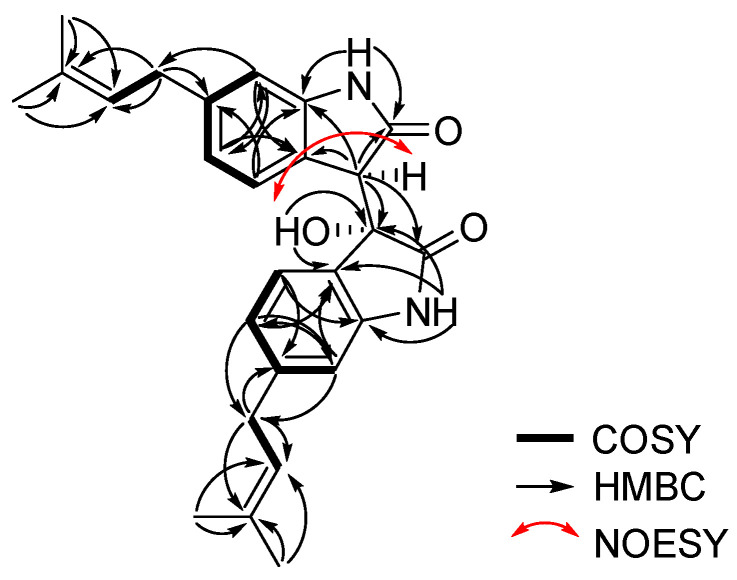
COSY, key HMBC and key NOESY correlations of saccharobisindole (**1**).

**Figure 3:**
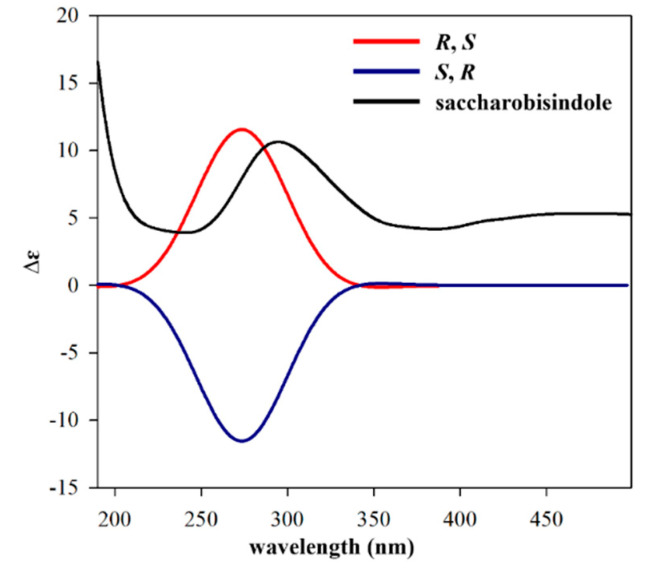
ECD spectra of saccharobisindole (**1**) and other isomers.

One set of indolin-2-one moieties with a 3-methylbut-2-enyl group was identified via analysis of HMBC correlations (Figure 2). HMBC correlations from H-10 to C-7 (*δ*_C_ 141.8), and from H-6 (*δ*_H_ 6.55) and H-8 (*δ*_H_ 6.80) to C-10 (*δ*_C_ 33.8) allowed for the attachment of a 3-methylbut-2-enyl group at C-7 of the benzene ring system. Further, HMBC correlations from the exchangeable NH-1 proton (*δ*_H_ 10.08) to C-5 (*δ*_C_ 143.4) and the carbonyl carbon C-2 (*δ*_C_ 174.6), and from the methine proton H-3 (*δ*_H_ 3.91) to C-2, C-4 (*δ*_C_ 123.2), and C-5, allowed for the assignment of an indolin-2-one moiety with a 3-methylbut-2-enyl group. Similarly, the other indolin-2-one moiety with a 3-methylbut-2-enyl group was also assigned by interpretation of HMBC correlations. Briefly, HMBC correlations of H-10′ (*δ*_H_ 3.19)/ C-7′ (*δ*_C_ 143.1); H-6′ (*δ*_H_ 6.53) and H-8′ (*δ*_H_ 6.45)/ C-10′ (*δ*_C_ 33.7); and NH-1′/ C-3′ (*δ*_C_ 75.4), C-4′ (*δ*_C_ 125.8), C-5′ (*δ*_C_ 142.9) provided further evidence for the assignment of an indolin-2-one moiety with a 3-methylbut-2-enyl group. A hydroxy group at *δ*_H_ 6.40 was attached to C-3′ based on HMBC correlations from this exchangeable proton to C-3′ and C-4′, as well as the consideration of the carbon chemical shift of C-3′ (*δ*_C_ 75.4). Lastly, the connection between C-3 and C-3′ of two indolin-2-one moieties was secured from the observation of HMBC correlations from H-3 to C-3′, thus completing the assignment of **1** (Figure 1). The relative configurations of **1** were deduced from the interpretation of nuclear Overhauser effect spectroscopy (NOESY) data. The NOESY correlation of **1** between H-3 and the 3′-OH protons indicated that the proton and hydroxy group should be placed at the same side of the rings (Figure 2). The absolute configuration of **1** was determined by calculating the experimental ECD data of **1** using density functional theory (DFT) modeling. The possible enantiomers of compound **1** were selected based on NOESY NMR data. The observed ECD spectrum of compound **1** showed a positive Cotton Effect around 291 nm. Comparison of the calculated ECD data of the two enantiomers with that measured, allowed the absolute configurations of compound **1** to be assigned as *R*, *S* (Figure 3). This result was also supported by comparing the experimental and calculated values of optical rotations. Compound **1** had a positive optical rotation value ${\left[\alpha \right]}_{D}^{25}\;$= +161). The calculated values of optical rotations for four theoretical stereoisomers indicated that three stereoisomers had all negative values and only the stereoisomer with 1R,1′S configurations displayed a positive value (${\left[\alpha \right]}_{D}^{25}\;$= +40). Therefore, **1** was assigned as 1R,1′S configuration (Figure S8). Compound **1** is an isatin derivative with structural differences at the side chains on two sets of indole moieties. Isatin itself was isolated from the plant *Cephalanceropsis gracilis* [13] and the deep-sea bacterium *Shewanella piezotolerans* [14].

Neoasterric methyl ester (**2**) was isolated as a yellowish oil and its molecular formula was assigned as C_17_H_16_O_8_ as determined by high-resolution fast atom bombardment mass spectrometry which illustrated a [M+H]^+^ *m/z* value of 348.0849 (calcd for C_17_H_16_O_8_, 348.0845), (HRFABMS). The IR absorptions at 3320 cm^−1^ and 1660 cm^−1^ indicated hydroxy and carbonyl functionalities, respectively. The ^1^H NMR spectrum showed bands for a 1,2,3,5-tetrasubstituted phenyl unit with two protons at H-2 (*δ*_H_ 5.77, 1H, s) and H-4 (*δ*_H_ 6.32, 1H, s), a 1,2,3,4-tetrasubstituted phenyl group at H-2′ (*δ*_H_ 6.64, 1H, d, *J* = 8.7 Hz) and H-3′ (*δ*_H_ 6.87, 1H, d, *J* = 8.7 Hz), two methoxy groups H-8 (*δ*_H_ 3.75, 3H, s) and H-8′ (*δ*_H_ 3.61, 3H, s), and one methyl singlet H-9 (*δ*_H_ 2.08, 3H, s). The ^1^H, ^13^C and HSQC spectroscopic data revealed one methyl C-9 (*δ*_C_ 21.4), two methoxys C-8 (*δ*_C_ 51.8) and C-8′ (*δ*_C_ 51.8), two carbonyls C-7 (*δ*_C_ 166.9) and C-7′ (*δ*_C_ 165.5), four aromatic methines C-2 (*δ*_C_ 105.1), C-4 (*δ*_C_ 109.7), C-2′ (*δ*_C_ 113.3), and C-3′ (*δ*_C_ 118.9), and eight quaternary carbons C-1 (*δ*_C_ 156.3), C-3 (*δ*_C_ 141.1), C-5 (*δ*_C_ 156.0), C-6 (*δ*_C_ 107.2), C-1′ (*δ*_C_ 142.2), C-4′ (*δ*_C_ 138.0), C-5′ (*δ*_C_ 147.7), and C-6′ (*δ*_C_ 116.6) (Table 1).

**Figure 4:**
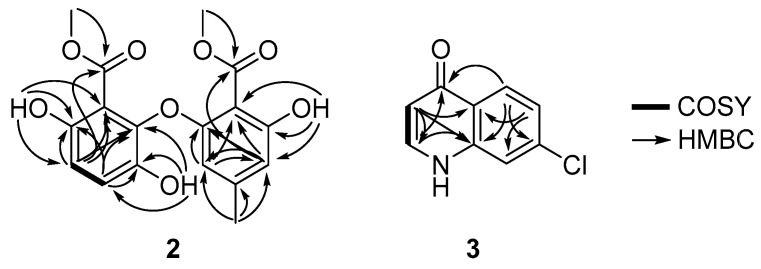
COSY and key HMBC correlations of neoasterric methyl ester (**2**) and 7-chloro-4(*1H*)-quinolone (**3**).

Analysis of COSY and HMBC spectroscopic data allowed the chemical structure of **2** to be assigned (Figure 4). HMBC correlations from H-2 (*δ*_H_ 5.77) to C-1 (*δ*_C_ 156.3), C-4 (*δ*_C_ 109.7), and C-6 (*δ*_C_ 107.2) and from H-4 (*δ*_H_ 6.32) to C-1 and C-2 (*δ*_C_ 105.1) supported the presence of a 1,2,3,5 substituted benzene moiety. Furthermore, the observation of HMBC correlations from the exchangeable proton 5-OH (*δ*_H_ 10.06) to C-4, C-5 (*δ*_C_ 156.0), and C-6 permitted the C-5/5-OH connectivity. HMBC correlations from the methyl singlet protons H-9 (*δ*_H_ 2.08) to C-2, C-3 (*δ*_C_ 141.1) and C-4 indicated that a methyl group was positioned at C-3. A methoxy group was then assigned as part of a methyl ester located at C-7 (*δ*_C_ 166.9) according to analysis of the three-bond HMBC correlation from H-8 (*δ*_H_ 3.75) to C-7. The long-range HMBC correlation from H-4 to C-6 (*δ*_C_ 107.2) and C-7 (*δ*_C_ 166.9) with the typical carbon chemical shift of C-6 (*δ*_C_ 107.2) suggested the attachment of C-6 to C-7. The ortho-coupled protons (H-2′ and H-3′, *J* *=* 8.7 Hz) displayed the COSY cross peaks H-2′/H-3′ and the HMBC correlations with C-1′ (*δ*_C_ 142.2), C-5′ (*δ*_C_ 147.7), and C-6′ (*δ*_C_ 116.0) and C-1′, C-4′ (*δ*_C_ 138.1), and C-5′, respectively. Based on these correlations, a 1,2,3,4 substituted aromatic moiety was established. The two exchangeable protons of 1′-OH (*δ*_H_ 9.56) and 4′-OH (*δ*_H_ 9.70) were placed at C-1′ and C-4′, respectively, based on the observation of HMBC correlations from 1′-OH to C-1′, C-2′ (*δ*_C_ 113.3), and C-6′ and from the 4′-OH to C-3′ (*δ*_C_ 118.9), C-4′, and C-5′. The HMBC correlation from the methoxy protons H-8′ to C-7′ (*δ*_C_ 165.5) suggested the presence of a methyl ester moiety and this functional group was placed at C-6′ (*δ*_C_ 116.6) based on the identification of a long-range HMBC correlation from H-2′ (*δ*_H_ 6.64) to C-6′. Lastly, the linkage of two aromatic rings through an oxygen atom (C-5′/O/C-1) was deduced from the typical oxygenated carbon chemical shifts of C-1 (*δ*_C_ 156.3) and C-5′ (*δ*_C_ 147.7). Compound **2** is an analog of asterric acid. Methyl asterrate, an asterric acid derivative, is most structurally similar to **2** except for the hydroxy group at C-4′ and the *ortho*-coupled protons (H-2′ and H-3′, *J* = 8.7 Hz) [15]. Methyl asterrate, also reported as trimethyllosoic acid, was isolated from fungi strains such as *Aspergillus* sp. [15], *Oospora* sp. [16], *Dothideomycete* sp. [17], *Phoma* sp. [18], *Pleosporales* sp. [19], *Preussia* sp. [20], and *Geomyces* sp. [21]. Methyl asterrate was found to display low cytotoxic activity in the HepG2 cell line with 22% growth inhibition at a 100 μg/mL concentration [17].

7-Chloro-4(*1H*)-quinolone (**3**) was isolated as a white amorphous powder and its chemical formula was assigned as C_9_H_6_^35^ClNO based on the observation of a protonated molecular ion peak at *m/z* 180.0218 [M+H]^+^ (calcd for C_9_H_6_^35^ClNO, 180.0216) in the HRFAB mass spectrum. The ^1^H NMR spectrum of **3** displayed two deshielded olefinic protons H-2 (*δ*_H_ 8.01, 1H, d, *J* = 7.3 Hz) and H-3 (*δ*_H_ 6.37, 1H, d, *J* = 7.3 Hz), and a 1,3,4-trisubstituted benzene ring with protons at H-5 (*δ*_H_ 8.24, 1H, d, *J* = 8.8 Hz) and H-6 (*δ*_H_ 7.42, 1H, dd, *J* = 8.8, 1.9 Hz), and H-8 (*δ*_H_ 7.63, 1H, d, *J* = 1.9 Hz). The ^13^C NMR spectrum exhibited nine carbons C-2 (*δ*_C_ 142.2), C-3 (*δ*_C_ 110.3), C-4 (*δ*_C_ 179.8), C-4a (*δ*_C_ 125.1), C-5 (*δ*_C_ 128.3), C-6 (*δ*_C_ 126.1), C-7 (*δ*_C_ 142.2), C-8 (*δ*_C_ 118.9), C-8a (*δ*_C_ 139.8). A quinolone moiety was identified based on the interpretation of COSY and HMBC spectroscopic data. The COSY correlation of H-2 (*δ*_H_ 8.01) with H-3 (*δ*_H_ 6.37) and long-range HMBC correlations from H-2 to C-4 (*δ*_C_ 179.8) indicated the presence of an α,β-unsaturated carbonyl group. Furthermore, three-bond HMBC correlations from H-2 to C-8a and from H-3 to C-4a, along with the carbon chemical shifts of C-2 (*δ*_C_ 142.2) and C-8a (*δ*_C_ 142.2), and correlations from H-6 to C-4a (*δ*_C_ 125.1) and C-8 (*δ*_C_ 118.9), and from H-5 to C-4, C-7 (*δ*_C_ 142.2) and C-8a (*δ*_C_ 139.8) allowed for the construction of the quinolone moiety. The carbon chemical shift at *δ*_C_ 142.2 and the isotope ratio (3:1) of two pseudomolecular ion peaks [M + H]^+^ and [M + H + 2]^+^ in the LR-ESI-MS spectroscopic data (Figure S19), allowed the attachment of a chlorine atom at C-7. Compound **3** was assigned as 7-chloro-4(*1H*)-quinolone (Figure 4). The structural assignment of **3** was completed by comparing the NMR data with the data from the literature [22].

Compound **3** has been reported as a synthesized product [22] but this is the first report from a natural source (i.e., a bacterium). Compound **3** was approved as an antitumor drug for the treatment of late mammary cancer and non-small cell lung cancer by the SFDA (State Food and Drug Administration of China) due to its capacity to damage DNA and block DNA synthesis in tumor cells [23]. Furthermore, **3** was found to exhibit weak inhibitory activity on cysteine protease [24].

In addition to **1**–**3**, four known natural products were isolated and identified as acremonidin E (**4**), pinselin (**5**), penicitrinone A (**6**), and penicitrinone E (**7**) by comparing their NMR and MS spectroscopic data with those of previously reported compounds. Interestingly, the known compounds were originally isolated from fungi and had never been reported to be produced by actinomycetes. Acremonidine E (**4**), penicitrinon A (**6**), and penicitrinon E (**7**) were isolated from *Penicillium* sp. [25,26,27], whereas pinselin (**5**) was previously identified in the endophytic fungus *Phomopsis* sp. [28], the marine fungus *Scopuariopsis* sp. [29], and the soil fungus *Penicillium* sp. [30]. In this study, a large-scale regrowth of the strain CNQ-490 allowed us to identify the minor compounds (**1**–**7**) produced by this strain as well as to rediscover the major compounds such as lodopyridones A–C, sacchromonopyrones A–C, and saccharoquinoline [7,8,9,10]. These data suggest that strain CNQ-490 has very good potential in producing biosynthetically-diverse secondary metabolites.

Compounds **1–7** were tested for their antibacterial activities against three Gram-positive bacteria (*Bacillus subtilis* KCTC1021, *Kocuria rhizophila* KCTC1915, and *Staphylococcus aureus* KCTC1927), and three Gram-negative bacteria (*Escherichia coli* KCTC2441, *Salmonella typhimurium* KCTC2515, and *Klebsiella pneumoniae* KCTC2690) (Table 2). In addition, quorum sensing (QS) inhibitory activity was evaluated with compounds **2**, and **4–7** against six pathogenic bacteria (*Cobetia marina* strain JEA023, *S. aureus* KCTC1927, *Micrococcus luteus* SCO560, *Pseudomonas aeruginosa* SNC165, *Pseudomonas fluorescens* SND204, and *Agrobacterium tumefaciens* SND195) (Table 3). The QS inhibitory activities of compounds **1** and **3** were not investigated, as these compounds could only be recovered in limited amounts. Compound **7** displayed strong antibacterial activity and weak QS inhibitory effect against *S. aureus* KCTC1927 with the values of 2.0 μg/mL and 64 μg/mL, respectively. Compound **2** performed weak inhibitory activities against *B. subtilis* KCTC1021 and *S. aureus* KCTC1927, with minimum inhibitory concentration (MIC) values of 32 μg/mL and 64 μg/mL, respectively. Furthermore, Compounds **5** and **6** exhibited strong QS blocking activities against *C. marina* JEA023 with the value 0.5 μg/mL and 1.0 μg/mL, respectively. Meanwhile, compounds **2**, **4**, and **6** exhibited QS blocking activities against *M. luteus* SCO560 at a 1–64 μg/mL range. None of the evaluated compounds exhibited QS inhibitory effects against *P. aeruginosa* SNC165, *P. fluorescens* SND204, and *A. tumefaciens* SND195.

## 3. Materials and Methods

### 3.1. General Experimental Procedures

UV spectra were recorded in MeOH on a Scinco UVS2100 spectrophotometer. IR spectra were collected using a Nicolet iS10 FT-IR spectrometer (Thermo Scientific Inc., Waltham, MA, USA). NMR spectra were obtained using an Agilent NMR spectrometer (Agilent, Santa Clara, CA, USA, at 400 for ^1^H and at 100 MHz for ^13^C) and a Bruker NMR spectrometer (Bruker, Middlesex, MA, USA, at 300 for ^1^H and and 75 MHz for ^13^C) using the signals of the residual solvent as internal references (*δ*_H_ 2.50 ppm and *δ*_C_ 39.5 ppm for dimethyl sulfoxide-*d_6_* (DMSO-*d_6_*) and *δ*_H_ 4.87 and 3.31 ppm and *δ*_C_ 49.1 ppm for deuterated methanol (CD_3_OD). Low-resolution LC/MS measurements were performed using the Agilent Technologies 1260 quadrupole and Waters Micromass ZQ LC/MS system using a reversed-phase column (Phenomenex Luna C18 (2) 100 Å, 50 mm × 4.6 mm, 5 μm) at a flow rate of 1.0 mL/min at the National Research Facilities and Equipment Center (NanoBioEnergy Materials Center) at Ewha Womans University. Open column chromatography was performed using silica (40–63 μm, Merck silica gel 60) eluting with a gradient solvent of dichloromethane (CH_2_Cl_2_) and methanol (MeOH). The fractions were purified via semi-preparative HPLC using a Waters 996 Photodiode Array Detected HPLC coupled with a reversed-phase Phenomenex Luna C18 (2) (100 Å, 250 nm × 10 mm, 5µm) column at a 2.0 mL/min flow rate. High-resolution mass spectra were recorded on a JMS-700 mass spectrometer (JEOL Ltd., Tokyo, Japan) at Seoul National University.

### 3.2. Strain Isolation and Fermentation

Strain CNQ-490 is an actinomycete that was isolated from a marine sediment sample obtained at a depth of 45 m from a submarine canyon in La Jolla, CA. Strain CNQ-490 was assigned to the genus *Saccharomonospora* based on 16S ribosomal DNA gene sequence similarity analyses and BLAST searches (GeneBank accession number EU214929). A total of 80 L of CNQ-490 was cultured in 20 × 2.5 L Ultra-Yield flasks (Thomson Scientific, Oceanside, CA, USA) each containing 1 L of the medium (10 g/L soluble starch, 2 g/L yeast, 4 g/L peptone, 10 g/L CaCO_3_, 20 g/L KBr, 8 g/L Fe_2_(SO_4_)_3_·4H_2_O dissolved in 750 mL natural seawater and 250 mL of distilled water) at 27 °C with constant shaking at 150 rpm. After 7 days of cultivation, the broth was extracted with ethyl acetate (EtOAc, 80 L overall) and the EtOAc-soluble fraction was dried in vacuo to obtain 6.5 g of organic extract.

### 3.3. Purification

The organic extract of CNQ-490 was fractionated by reversed-phase C-18 flash vacuum chromatography eluting with a step gradient from 0 to 100% methanol in water, which resulted in 11 fractions. Both fractions 3 and 4 (eluted with 9% and 10% MeOH in water, respectively) were further purified by HPLC (Phenomenex 100 Å, 250 nm × 10 mm, 5 µm, UV = 285 nm), with 60% of acetonitrile in H_2_O at a flow rate of 2.0 mL/min to yield **1** (1.2 mg, t_R_ 33.7 min), 45% of acetonitrile in H_2_O at a flow rate 2.0 mL/min to yield **2** (4.1 mg, t_R_ 27.5 min), **4** (40.3 mg, t_R_ 17.0 min), **5** (6.0 mg, t_R_ 40.0 min), **6** (4.8 mg, t_R_ 18.0 min), and **7** (6.7 mg, t_R_ 35.5 min), and 25% of acetonitrile in H_2_O at flow rate 2.0 mL/min to yield **3** (1.7 mg, t_R_ 33.6 min).

Saccharobisindole (**1**): Pale yellow oil, ${\left[\alpha \right]}_{D}^{25}\;$= +161 (*c* 0.29, MeOH), UV (MeOH) λ_max_ (log ε) 202 (2.93), 253 (2.64), 271 (2.08) nm; IR (KBr) ν_max_ 3431, 2935, 1676, 1638, 1444, 1196, 994 cm^−1^, ^1^H and ^13^C NMR data: Table 1 and Figures S1–S5; HR-FAB-MS *m/z* 417.2181 [M+H]^+^ (calculated for C_26_H_29_N_2_O_3_, 417.2178).

Neoasterric methyl ester (**2**): Yellowish oil, UV (MeOH) λ_max_ (log ε) 216 (2.42), 262 (1.93), 397 (1.65) nm; IR (KBr) ν_max_ 3320, 1660, 1616, 1439, 1314, 1201, 1077 cm^−1^, ^1^H and ^13^C NMR data: Table 1 and Figures S8–S12; HR-FA-BMS *m/z* 348.0849 [M+H]^+^ (calculated for C_17_H_16_O_8_, 348.0845).

7-Chloro-4(1H)-quinolone (**3**): White amorphous powder, UV (MeOH) λ_max_ (log ε) 218 (2.58), 278 (1.87), 279 (1.77) nm; IR (KBr) ν_max_ 3326, 1729, 1457, 802 cm^−1^, ^1^H and ^13^C NMR data: Table 1 and Figures S13–S17; HR-FAB-MS m/z 180.0218 [M+H]^+^ (calculated for C_9_H_6_^35^ClNO, 180.0216).

Acremonidine E (**4**): ^1^H NMR: (400 MHz, DMSO-*d_6_*); *δ*_H_ 11.36 (s, 2-OH), 11.36 (s, 6-OH), 9.70 (s, 1′-OH), 9.18 (s, 4′-OH), 6.94 (d, *J* = 8.8 Hz, H-5′), 6.80 (d, *J* = 8.8 Hz, H-6′), 6.10 (s, H-5), 6.10 (s, H-6), 3.54 (s, H-8′), 2.16 (s, H-7), ^13^C (100 MHz, DMSO-*d_6_*); *δ*_C_ 180.2 (C-8), 167.9 (C-7′), 161.5 (C-2), 161.5 (C-6), 151.1 (C-1′). 147.7 (C-4), 145.6(C-4′), 131.2 (C-3′), 122.1 (C-5′), 117.4 (C-6′), 112.3 (C-2′), 108.9 (C-1), 107,4 (C-3), 107.4 (C-5), 51.9 (C-8′), 21.6 (C-7), LR-ESI-MS *m/z* 319.1 [M+H]^+^.

Pinselin (**5**): ^1^H NMR:(400 MHz, DMSO-*d_6_*); *δ*_H_ 12.18 (s, 2-OH), 10.47 (s, 1′-OH), 7.45 (d, *J* = 9.1 Hz, H-5′), 7.60 (d, *J* = 9.1 Hz, H-6′), 6.89 (s, H-5), 6.65 (s, H-3), 3.84 (s, H-8′), 2.39 (s, H-7); ^13^C (100 MHz, DMSO-*d_6_*); *δ*_C_ 180.2 (C-8), 166.8 (C-7′), 160.4 (C-2), 155.4 (C-6), 150.7 (C-1′), 149.3 (C-4), 148.8 (C-4′), 125.3 (C-5′), 120.1 (C-6′), 117.2 (C-3′), 117.0 (C-2′), 110.7 (C-3), 107.4 (C-5), 106.0 (C-1), 52.2 (C-8′), 22.0 (C-7), LR-ESI-MS *m/z* 301.1 [M+H]^+^.

Penicitrinon A (**6**): ^1^H NMR: (400 MHz, CD_3_OD); *δ*_H_ 8.36 (s, 6′-OH), 6.68 (s, H-7), 5.38 (q, *J* = 6.8 Hz, H-3), 4.71 (dq, *J* = 6.7 and 4.0 Hz, H-2′), 3.48 (q, *J* = 6.8 Hz, H-4), 3.36 (dq, *J* = 6.7 and 4.0 Hz, H-3′), 2.30 (s, 5′-CH_3_), 2.22 (s, 5-CH_3_), 1.45 (d, *J* = 6.8 Hz, 3-CH_3_), 1.42 (d, *J* = 6.7 Hz, 2′-CH_3_), 1.35 (d, *J* = 6.7 Hz, 3′-CH_3_), 1.34 (d, *J* = 6.8 Hz, 4-CH_3_), ^13^C (100 MHz, CD_3_OD); *δ*_C_ 177.8 (C-6), 167.5 (C-1), 160.3 (C-8), 150.7(C-6′), 145.6 (C-4′), 138.5 (C-7′a), 137.4 (C-5), 128.8 (C-4a), 120.1 (C-5′), 105.7 (C-7′), 102.7 (C-8a), 101.6 (C-7), 89.0 (C-2′), 86.3 (C-3), 46.3 (C-3′), 45.8 (C-4), 21.2 (2′-CH_3_), 19.5 (3′-CH_3_), 18.9 (3-CH_3_), 18.8 (4-CH_3_), 12.1(5′-CH_3_), 10.7 (5-CH_3_), LR-ESI-MS *m/z* 381.1 [M+H]^+^.

Penicitrinon E (**7**): ^1^H NMR: (400 MHz, CD_3_OD); *δ*_H_ 5.17 (q, *J* = 6.8 Hz, H-3), 4.77 (dq, *J* = 6.5 and 4.2 Hz, H-2′), 3.29 (q, *J* = 6.8 Hz, H-4), 3.25 (dq, *J* = 6.5 and 4.2 Hz, H-3′), 2.28 (s, 5′-CH_3_), 2.22 (s, 5-CH_3_), 1.49 (d, *J* = 6.8 Hz, 3-CH_3_), 1.47 (d, *J* = 6.7 Hz, 2′-CH_3_), 1.38 (d, *J* = 7.0 Hz, 4-CH_3_), 1.36 (d, *J* = 7.0 Hz, 3′-CH_3_), LR-ESI-MS *m/z* 425.1 [M+H]^+^.

### 3.4. Minimum Inhibitory Concentration (MIC) against Gram-Positive and Gram-Negative Bacteria

For the antibacterial susceptibility assays, compounds **1**–**7** were tested against three Gram-positive bacteria (*B. subtilis* KCTC1021, *K. rhizophila* KCTC1915, and *S. aureus* KCTC1927) and three Gram-negative bacteria (*E. coli* KCTC2441, *S. typhimurium* KCTC2515, and *K. pneumoniae* KCTC2690) following the recommendations of previous studies. The bacterial inoculum was prepared in Muller-Hinton broth at 37 °C and 225 rpm for 24 h. The stock solution of compounds **1**–**7** and the positive control were dissolved at a 10 mg/mL concentration in DMSO and diluted with Muller-Hinton broth to obtain a 0.25–256 µg/mL concentration range. Bacterial inoculum (5 × 10^5^ CFU/mL concentration) was then dispensed into each well. The resulting mixtures consisting of varying concentrations of compounds **1**–**7** with a 1 × 10^6^ CFU/mL concentration of bacterial inoculum were transferred to 96 well microtiter plates and incubated at 37 °C for 24 h.

### 3.5. Detection of Anti-Quorum Sensing Activity

The 96-well plate method was employed to detect the anti-quorum sensing activity of different compounds against six bacterial strains (*C. marina* JEA023, *M. luteus* SCO560, *S. aureus* KCTC1927, *P. aeruginosa* SNC165, *P. fluorescens* SNA239, and *A. tumefaciens* SND195). *S. aureus* KCTC1927 and *A. tumefaciens* SND195 were cultured in tryptic soy broth (TSB), whereas *P. aeruginosa* SNC165 and *P. fluorescens* SND204 were cultured in King’s broth (KB). *C. marina* JEA023 and *M. luteus* SCO560 were cultured in marine broth maintaining a 0.5 McFarland standard turbidity (1.0 × 10^8^ CFU/mL). Dimethyl sulfoxide (100% DMSO) was used as a negative control, and kanamycin and rifampin (10 mg/mL each) were used as a positive control. Next, 50 µL of inoculum was inoculated into each well and 10 mg/mL of compound **2** and **4**–**7** dissolved in DMSO was diluted with marine broth media to produce concentrations ranging from 0.25 to 256 µg/mL. The mixtures of compounds and broth were dispensed into each inoculated well. The 96-well plates were then incubated for 16–18 h at 37 °C depending on the bacterial strains.

### 3.6. Conformational Analysis and ECD Spectrum Calculations

A conformational analysis of saccharobisindole (**1**) was carried out by MacroModel with the Merck molecular force field (gas phase), a 10 kJ/mol upper energy limit, and a 0.001 kJ (mol Å)^−1^ convergence threshold on the rms gradient to minimize computational complexity and expense. The possible enantiomers of **1** were selected based on NOESY NMR data and the energy-minimized enantiomer structures were generated by Avogadro 1.2.0. Energy minimization of the two structures was performed by Turbomole X 4.3.2. The calculated ECD spectra corresponding to two enantiomer models were calculated using DFT at the functional B3LYP/DFT level and the def-SV(P) basis set. The ECD spectra were simulated by overlapping each transition, where *σ* is the width of the band at 1/*e* height. ∆*Ei* is the excitation energies and *Ri* is rotatory strengths for transition *i*. In this calculation, the *σ* value was at 0.10 eV. The observed ECD spectrum of compound **1** showed positive cotton effect around 291 nm. Comparing the calculated spectra of the two enantiomers with the measured ECD spectrum, the absolute configurations of compound **1** were deduced as *R*, *S*.

$$\Delta \epsilon \left(E\right)=\frac{1}{2.297\times 10^{-39}}\frac{1}{\sqrt{2\pi \sigma }}\overset{A}{\underset{i}{\sum }}\Delta E_{i}R_{i}e^{[-{\left(E-\Delta E_{i}\right)}^{2}/\left(2\sigma {)}^{2}\right]}$$

## 4. Conclusions

The identification of the chemical components produced by strain CNQ-490 has led to the discovery of the new compounds saccharobisindole (**1**), neoasterric methyl ester (**2**), and 7-chloro-4(*1H*)-quinolone (**3**), in addition to four known compounds. Compound **1** is an isatin derivative and **2** contains two substituted benzene methyl esters with an ether linkage. Compound **3** possesses a chloroquinolone moiety and our study was the first to isolate this compound from a natural source. Additionally, our study was the first to isolate compounds **4**–**7**, reported as fungal metabolites, from a bacterial strain. Compound **2** exhibited antibacterial activity against *B. subtilis* KCTC 1021 and *S. aureus* KCTC 1927, as well as a weak QS inhibitory activity against *S. aureus* KCTC 1927.

## Figures and Tables

**Table 1:** NMR spectroscopic data for saccharobisindole (**1**) and neoasterric methyl ester (**2**) in DMSO-*d*_6_^1^, and 7-chloro-4(*1H*)-quinolone (**3**) in CD_3_OD ^2^.

No.	Saccharobisindole (1)					Neoasterric Methyl Ester (2)				7-Chloro-4(*1H*)-quinolone (3)		
	*δ*C, Type ^3^	*δ*H, (*J* in Hz)	COSY	HMBC	*δ*C, Type ^3^	*δ*H, (*J* in Hz)	COSY	HMBC	*δ*C, Type ^3^	*δ*H, (*J* in Hz)	COSY	HMBC
1	NH	10.08, s		C-2, 3, 4, 5	156.3, C							
2	174.6, C				105.1, CH	5.77, s		C-1, 4, 6	142.2, CH	8.01, d (7.3)	H-3	C-3, 4, 8a
3	51.1, CH	3.91, s		C-2, 4, 5, 2′, 3′, 4′	141.1, C				110.3, CH	6.37, d (7.3)	H-2	C-2, 4a
4	123.2, C				109.7 CH	6.32, s		C-1, 2, 6, 7	179.8, C			
4a									125.1, C			
5	143.4, C				156.0, C				128.3, CH	8.24, d (8.8)	H-6	C-4, 7, 8, 8a
6	108.8, CH	6.55, s	H-8	C-4, 8, 10	107.2, C				126.1, CH	7.42, dd (8.8, 1.9)	H-5	C-4a, 8, 8a
7	141.8, C				166.9, C				142.2 C			
8	120.9, CH	6.80, d (7.7)	H-6, 9	C-4, 6, 10	51.8, CH3	3.75, s		C-7	118.9, CH	7.63, d (1.9)		C-4a, 6, 8a
8a									139.8, C			
9	126.2, CH	7.35, d (7.7)	H-8	C-5, 7	21.4, CH3	2.08, s	2, 4	C-2, 3, 4				
10	33.8, CH2	3.30, d (7.9)	H-11	C-6, 7, 8, 11, 12								
11	123.2, CH	5.29, t (7.9)	H-10	C-10, 13, 14								
12	131.8, C											
13	25.5, CH3	1.72, s		C-11, 12, 14								
14	17.7, CH3	1.69, s		C-11, 12, 13								
1′	NH	10.22, s		C-3′, 4′, 5′	142.2, C							
2′	177.3, C				113.3, CH	6.64, d (8.7)	3′	C-1′, 5′, 6′,				
3′	75.4, C				118.9, CH	6.87, d (8.7)	2′	C-1′, 4′, 5′				
4′	125.8, C				138.0, C							
5′	142.9, C				147.7, C							
6′	109.4, CH	6.53, s	H-8′	C-4′, 8′, 10′	116.6, C							
7′	143.1, C				165.5, C							
8′	120.8, CH	6.45, d (7.6)	H-9′	C-4′, 6′, 10′	51.8, CH3	3.61, s		C-7′				
9′	123.5, CH	6.09, d (7.6)	H-6′, 8′	C-5′, 7′								
10′	33.7, CH2	3.19, d (7.5)	H-11′	C-6′, 7′, 8′, 11′, 12′								
11′	122.9, CH	5.21, t (7.5)	H-10′	C-10′, 13′, 14′								
12′	131.9, C											
13′	25.4, CH3	1.68, s		C-11′, 12′, 14′								
14′	17.6, CH3	1.64, s		C-11′, 12′, 13′								
5-OH					10.06, s			C-4, 5, 6				
1′-OH					9.56, s			C-1′, 2′, 6′				
3′-OH		6.40, s		C-3, 3′, 4′								
4′-OH					9.70, s			C-3′, 4′, 5′				

^1^ 300 MHz for ^1^H NMR and 75 MHz for ^13^C NMR.; ^2^ 400 MHz for ^1^H NMR and 100 MHz for ^13^C NMR.; ^3^ Multiplicity was determined by the analysis of 2D NMR spectroscopic data.

**Table 2:** Minimum inhibitory concentration (MIC) of **1**–**7** against Gram-positive and Gram-negative bacterial strains.

Compound	MIC (μg/mL)					
	Gram (+) Bacteria			Gram (−) Bacteria		
	*B. subtilis* KCTC1021	*K. rhizophila* KCTC1915	*S. aureus* KCTC1927	*E. coli* KCTC2441	*S. typhimurium* KCTC2515	*K. pneumonia* KCTC2690
**1**	>128	>128	>128	>128	>128	>128
**2**	32	>128	64	>128	>128	>128
**3**	>128	>128	>128	>128	>128	>128
**4**	>128	>128	>128	>128	>128	>128
**5**	>128	>128	>128	>128	>128	>128
**6**	>128	>128	>128	>128	>128	>128
**7**	>128	>128	2	>128	>128	>128
Ampicillin	1	0.5	2	16	16	>128
Vancomycin	0.25	1	1	>128	>128	>128

**Table 3:** Anti-quorum sensing activity of **2**, **4**–**7**.

Compound	Anti-Quorum Sensing (μg/mL)					
	Bacteria					
	*C. marina* JEA023	*S. aureus* KCTC1927	*M. luteus* SCO560	*P. aeruginosa* SNC165	*P. fluorescens* SND204	*A. tumefaciens* SND195
**2**	>128	32	64	>128	>128	>128
**4**	>128	16	32	>128	>128	>128
**5**	0.5	64	>128	>128	>128	>128
**6**	16	8	1	>128	>128	>128
**7**	>128	64	>128	>128	>128	>128
Kanamycin	2	8	16	>128	16	64
Rifampin	8	0.25	0.25	4	4	1

## Data Availability

The data presented in this study are available on request from the corresponding author.

## Supplementary Materials

The following are available online at https://www.mdpi.com/article/10.3390/md20010035/s1, Figures S1–S8: 1D and 2D NMR, high resolution mass data, & calculated optical rotation of compound **1**; Figures S9–S13: 1D and 2D NMR data of compound **2**; Figures S14–S19: 1D and 2D NMR, and LR-ESI-MS spectroscopic data of compound **3**; Figures S20–S26: ^1^H and ^13^C NMR of compounds **4**–**7**.
