# Supplementary material for: Saccharobisindole, Neoasterric Methyl Ester, and 7-Chloro-4(1H)-quinolone: Three New Compounds Isolated from the Marine Bacterium Saccharomonospora sp
Source: Mar Drugs. 2021 Dec 29;20(1):35. doi: 10.3390/md20010035 (PMC8778701; doi:10.3390/md20010035)

Supporting Information

Supplementary File

Saccharobisindole, neoasterric methyl ester, and 7-chloro-4(*1H*)-quinolone: three new compounds isolated from the marine bacterium *Saccharomonospora* sp.

Sohee Kim[1,†], Tu Cam Le[2,3,†], Sang-Ah Han[1,†], Prima F. Hillman[4], Ahreum Hong[1,5], Sunghoon Hwang[6], Young Eun Du[6], Hiyoung Kim[7], Dong-Chan Oh[6], Sun-Shin Cha[4], Jihye Lee[4,5,], Sang-Jip Nam[4,*], William Fenical[8,*]*

[1] The Graduate School of Industrial Pharmaceutical Sciences, Ewha Womans University, Seoul 03760, Republic of Korea

[2] Laboratory of Advanced Materials Chemistry, Advanced Institute of Materials Science, Ton Duc Thang University, Ho Chi Minh City, Vietnam

[3] Faculty of Applied Sciences, Ton Duc Thang University, Ho Chi Minh City, Vietnam

[4] Department of Chemistry and Nanoscience, Ewha Womans University, Seoul 03760, Republic of Korea

[5] Laboratories of Marine New Drugs, REDONE Seoul, Seoul 08594, Republic of Korea

[6] Natural Products Research Institute, College of Pharmacy, Seoul National University, Seoul 08826, Republic of Korea;

[7] Department of Biomedical Science and Engineering, Konkuk University, Seoul 05029, Republic of Korea

[8] Center for Marine Biotechnology and Biomedicine, Scripps Institution of Oceanography, University of California-San Diego, La Jolla, CA 92093-0204, USA

***** Correspondence: jl3414@gmail.com; Tel.: +82-70-4219-1632 (J.L.); sjnam@ewha.ac.kr; Tel.: +82-2-3277-6805 (S.-J.N.); wfenical@ucsd.edu (W.F.); Tel: +1-858-259-3839 (W.F.)

List of Supplementary Content

Figure S1. ^{1}H NMR spectrum (300 MHz) of sacharobisindole (**1**) in DMSO-d_6 …………………………………………. S4

Figure S2. ^{13}C NMR spectrum (75 MHz) of sacharobisindole (**1**) in DMSO-d_6 …………………………………………. S5

Figure S3. COSY spectrum (300 MHz) of sacharobisindole (**1**) in DMSO-d_6 ……………………………………………. S6

Figure S4. HSQC spectrum (300 MHz) of sacharobisindole (**1**) in DMSO-d_6 ……………………………………………. S7

Figure S5. HMBC spectrum (300 MHz) of sacharobisindole (**1**) in DMSO-d_6……………………………………...………. S8

Figure S6. NOESY spectrum (400 MHz) of sacharobisindole (**1**) in DMSO-d_6…………………………………...…………. S9

Figure S7. High resolution mass data of sacharobisindole (**1**) ……………………………………………………...……. S10

Figure S8. Alpha-D data and calculated optical rotation of saccharobisinodle (**1**)……………………………..……..…. S11

Figure S9. ^{1}H NMR spectrum (400 MHz) of neoasterric methyl ester (**2**) in DMSO-d_6………………………………. S12

Figure S10. ^{13}C NMR spectrum (100 MHz) of neoasterric methyl ester (**2**) in DMSO-d_6…………………...……. S13

Figure S11. COSY spectrum (400 MHz) of neoasterric methyl ester (**2**) in DMSO-d_6……………………...……. S14

Figure S12. HSQC spectrum (400 MHz) of neoasterric methyl ester (**2**) in DMSO-d_6………………………………. S15

Figure S13. HMBC spectrum (400 MHz) of neoasterric methyl ester (**2**) in DMSO-d_6……………………………….. S16

Figure S14. ^{1}H NMR spectrum (400 MHz) of 7-chloro-4(*1H*)-quinolone (**3**) in CD_3OD…………………………….. S17

Figure S15. ^{13}C NMR spectrum (100 MHz) of 7-chloro-4(*1H*)-quinolone (**3**) in CD_3OD …………………………….. S18

Figure S16. COSY spectrum (400 MHz) of 7-chloro-4(*1H*)-quinolone (**3**) in CD_3OD ……………………………….. S19

Figure S17. HSQC spectrum (400 MHz) of 7-chloro-4(*1H*)-quinolone (**3**) in CD_3OD ……………………………….. S20

Figure S18. HMBC spectrum (400 MHz) of 7-chloro-4(*1H*)-quinolone (**3**) in CD_3OD ……………………………….. S21

Figure S19. LR-ESI-MS spectroscopic data of 7-chloro-4(1H)-quinolone (**3**)……………………………………… S22

Figure S20. ^{1}H NMR spectrum (400 MHz) of acremonidine E (**4**) in DMSO-d_6……………………………………… S23

Figure S21. ^{13}C NMR spectrum (100 MHz) of acremonidine E (**4**) in DMSO-d_6……………………………………… S24

Figure S22. ^{1}H NMR spectrum (400 MHz) of pinselin (**5**) in DMSO-d_6……………………………………………… S25

Figure S23. ^{13}C NMR spectrum (100 MHz) of pinselin (**5**) in DMSO-d_6…………………………………………… S26

Figure S24. ^{1}H NMR spectrum (400 MHz) of penicitrinon A (**6**) in CD_3OD S27
Figure S25. ^{13}C NMR spectrum (100 MHz) of penicitrinon A (**6**) in CD_3OD S28
Figure S26. ^{1}H NMR spectrum (400 MHz) of penicitrinon E (7) in CD_3OD S29

Figure S1. ^{1}H NMR spectrum (300 MHz) of saccharobisindole (**1**) in DMSO-d_6

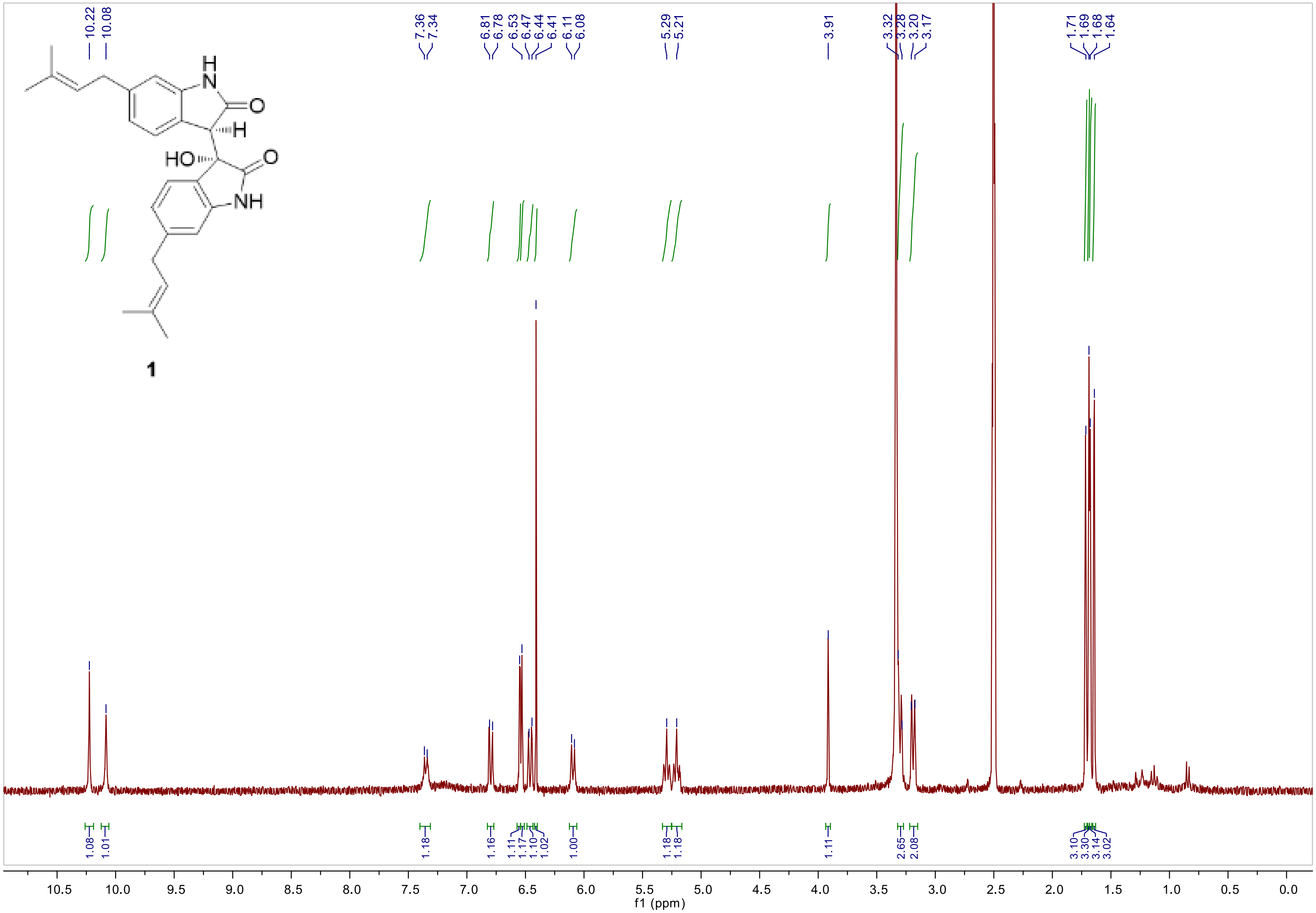

Figure S2. ^{13}C NMR spectrum (75 MHz) of saccharobisindole (**1**) in DMSO-d_6

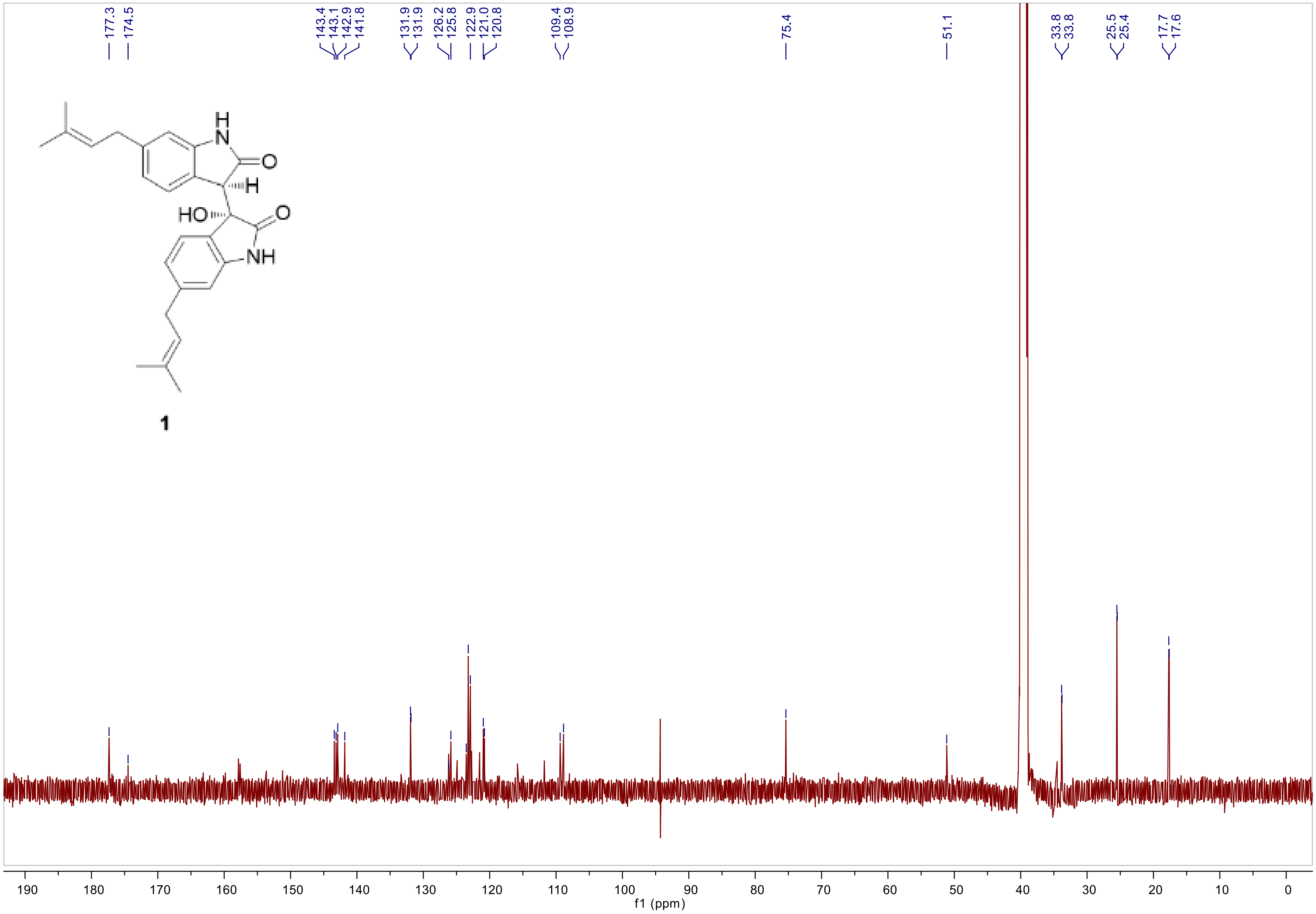

Figure S3. COSY spectrum (300 MHz) of saccharobisindole (**1**) in DMSO-d_6

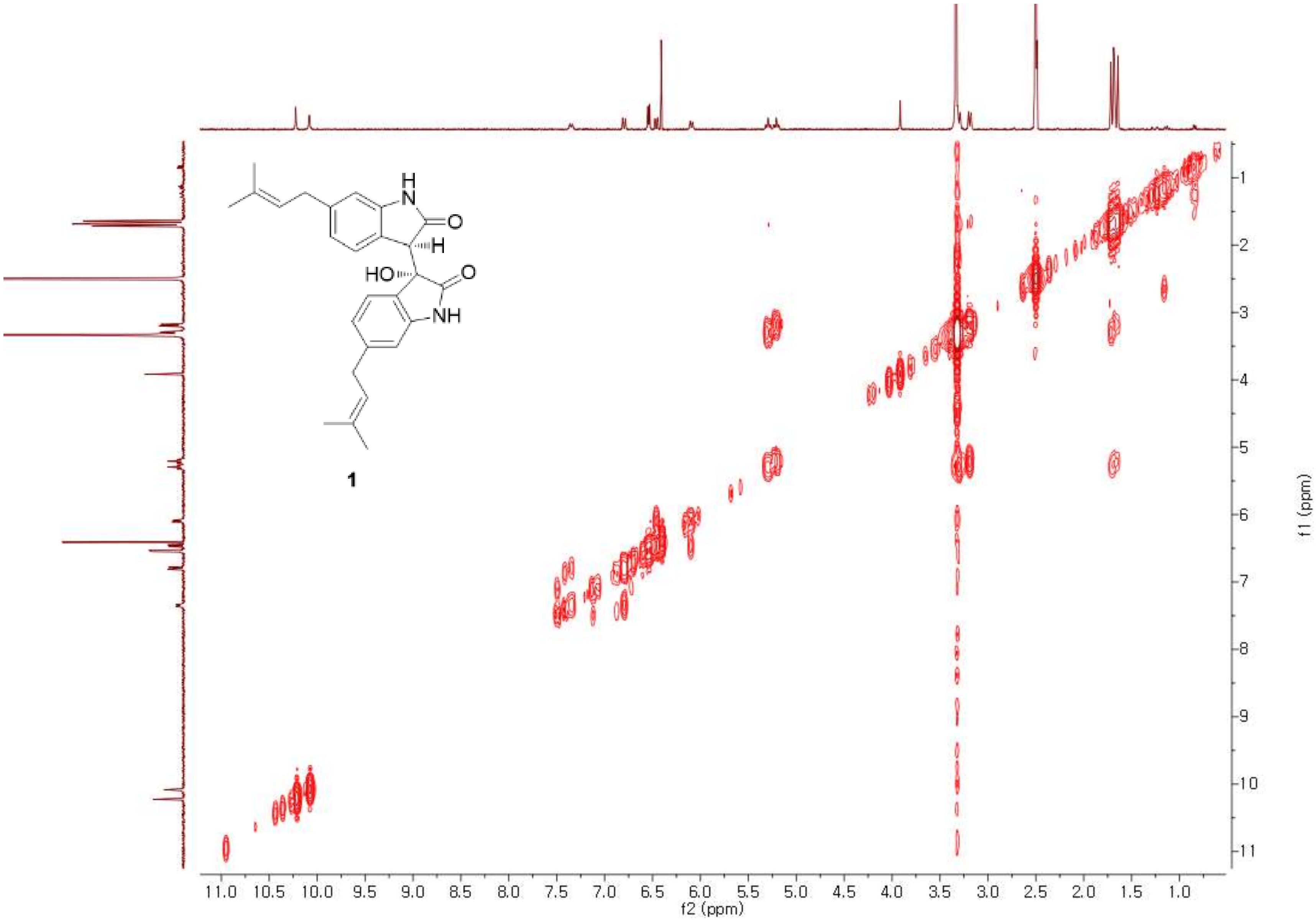

Figure S4. HSQC spectrum (300 MHz) of saccharobisindole (**1**) in DMSO-d_6

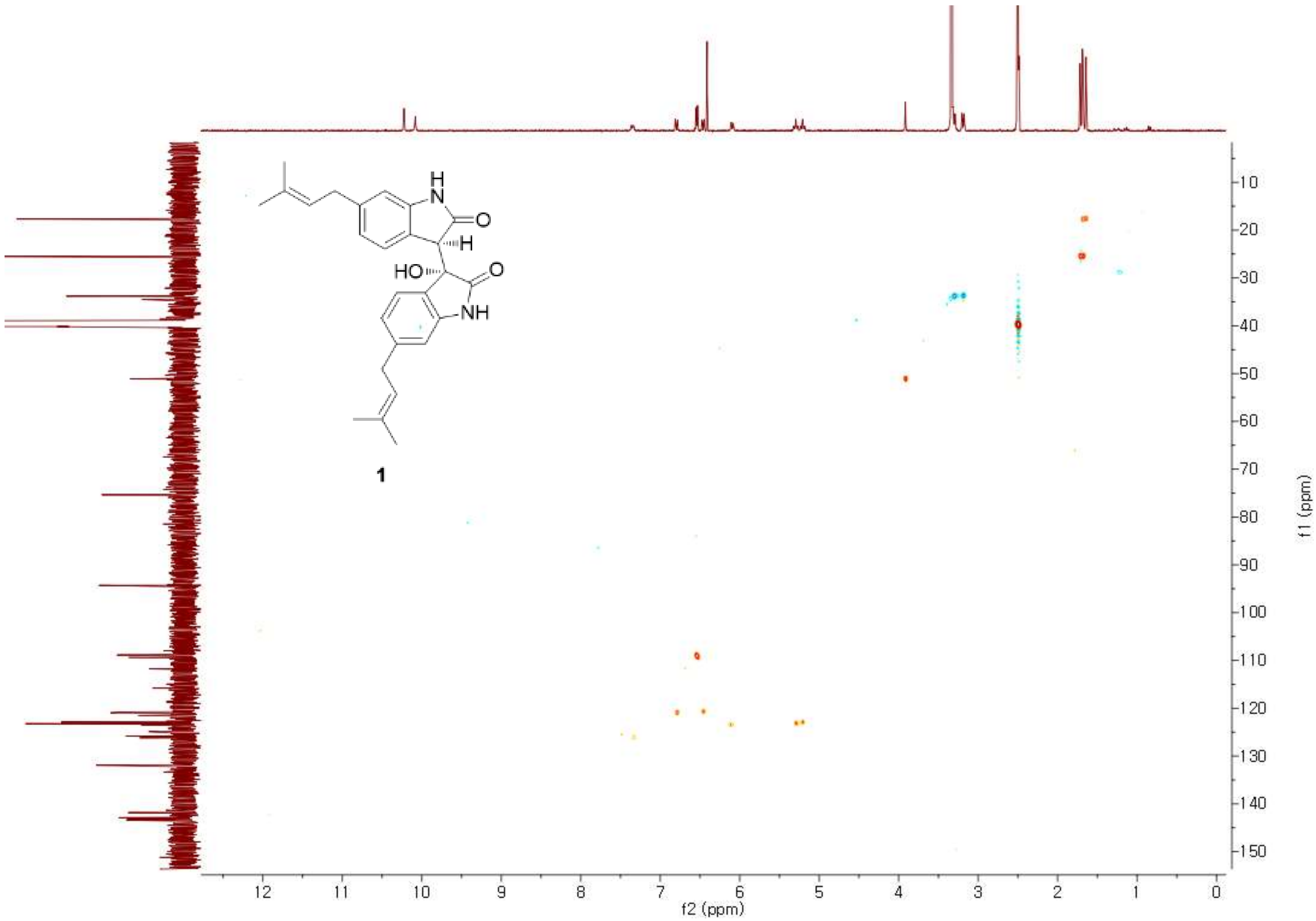

Figure S5. HMBC spectrum (300 MHz) of saccharobisindole (**1**) in DMSO-d_6

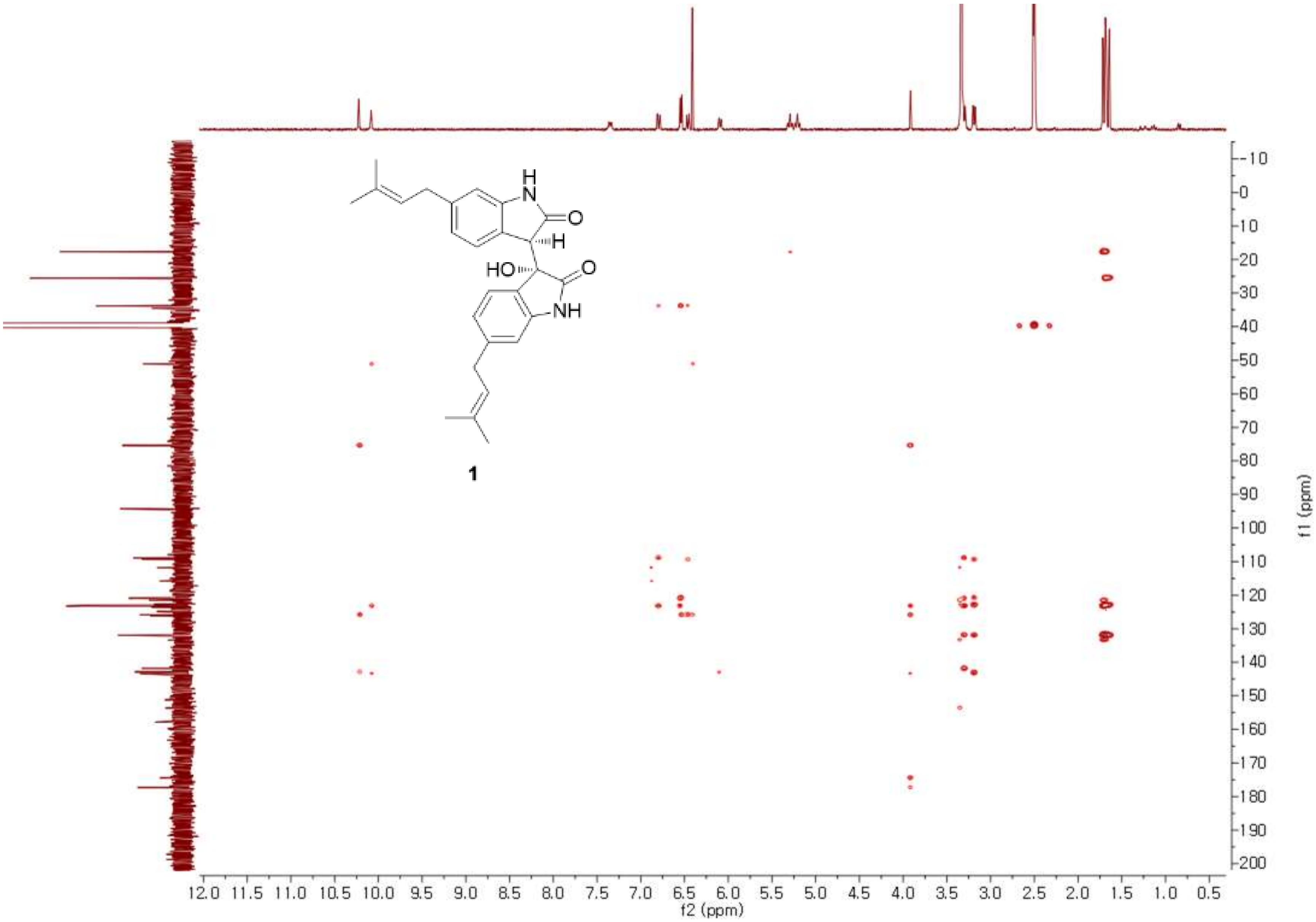

Figure S6. NOESY spectrum (300 MHz) of saccharobisindole (**1**) in DMSO-d_6

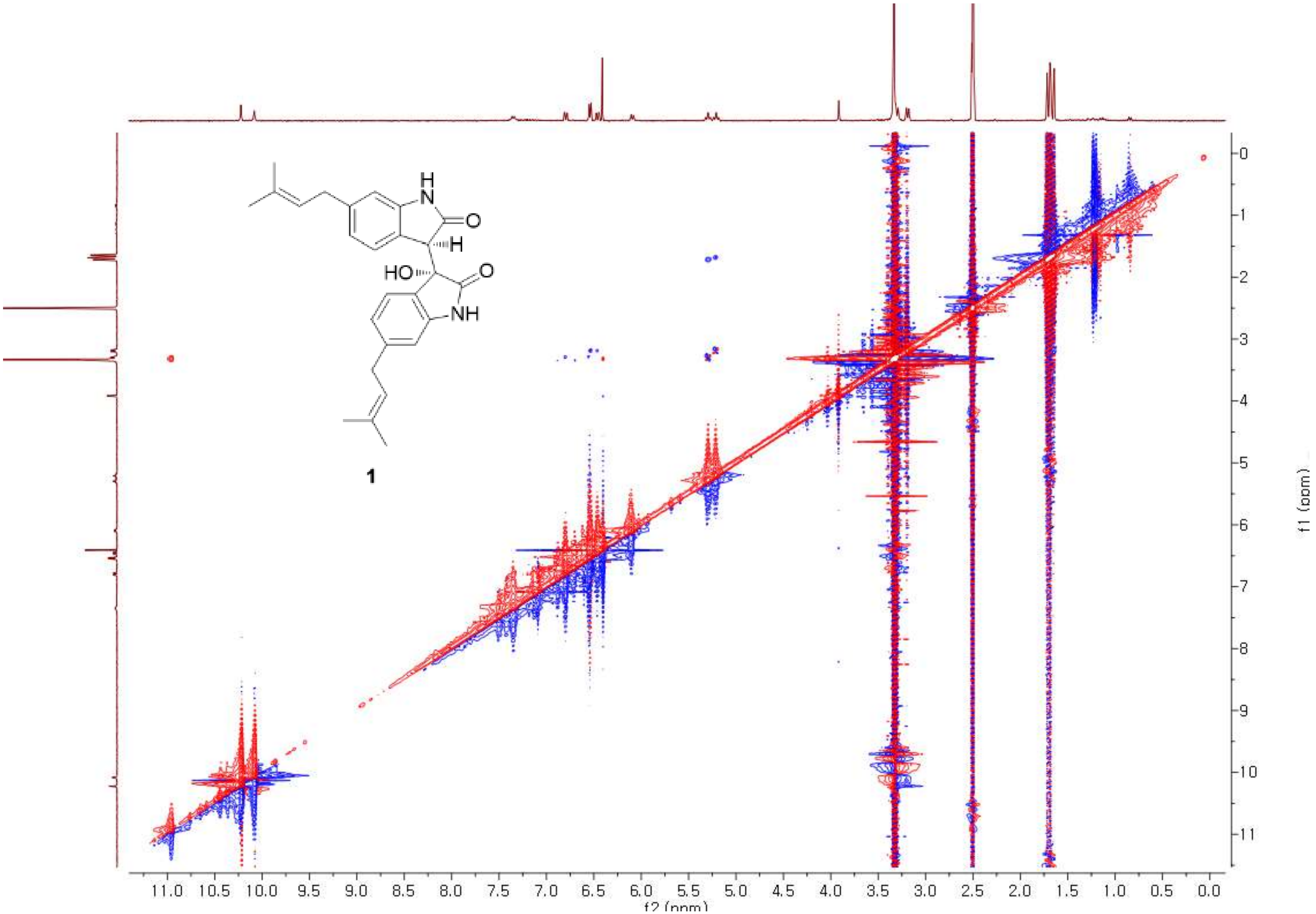

Figure S7. High resolution mass data of saccharobisindole (**1**)

```
[ Elemental Composition ]
Data   : FAB-P861                    Date : 29-Sep-2017 17:17
Sample: CNQ490D5-1(C5-1)
Note : m-NBA
Inlet : Direct                       Ion Mode : FAB+
RT : 1.81 min                        Scan#: (81,88)
Elements : C 100/0, H 100/0, N 10/0, O 10/0
Mass Tolerance     : 20ppm, 5mmu if m/z < 250, 10mmu if m/z > 500
Unsaturation (U.S.) : -0.5 - 50.0
```

Observed m/z	Int%	Err[ppm / mmu]	U.S.	Composition
417.2181	0.6	-5.7 / -2.4	18.0	C 29 H 27 N 3
		-19.8 / -8.3	14.5	C 21 H 25 N 10
		-8.9 / -3.7	17.5	C 31 H 29 O
		+7.1 / +3.0	14.5	C 22 H 25 N 8 O
		+3.9 / +1.6	14.0	C 24 H 27 N 5 O 2
		+0.7 / +0.3	13.5	C 26 H 29 N 2 O 3
		-13.4 / -5.6	10.0	C 18 H 27 N 9 O 3
		+16.8 / +7.0	10.5	C 17 H 25 N 10 O 3
		-16.6 / -6.9	9.5	C 20 H 29 N 6 O 4
		+13.6 / +5.7	10.0	C 19 H 27 N 7 O 4
		-19.8 / -8.3	9.0	C 22 H 31 N 3 O 5
		+10.3 / +4.3	9.5	C 21 H 29 N 4 O 5
		+7.1 / +3.0	9.0	C 23 H 31 N O 6
		-6.9 / -2.9	5.5	C 15 H 29 N 8 O 6
		-10.2 / -4.2	5.0	C 17 H 31 N 5 O 7
		+20.0 / +8.3	5.5	C 16 H 29 N 6 O 7
		-13.4 / -5.6	4.5	C 19 H 33 N 2 O 8
		+16.8 / +7.0	5.0	C 18 H 31 N 3 O 8
		+2.7 / +1.1	1.5	C 10 H 29 N 10 O 8
		+13.5 / +5.7	4.5	C 20 H 33 O 9
		-0.5 / -0.2	1.0	C 12 H 31 N 7 O 9
		-3.7 / -1.6	0.5	C 14 H 33 N 4 O 10

```
[ Theoretical Ion Distribution ]
Molecular Formula : C26 H29 N2 O3
                        (m/z   417.2178, MW   417.5279, U.S. 13.5)
Base Peak :   417.2178, Averaged MW :   417.5252(a),   417.5260(w)

   m/z        INT.
 417.2178 100.0000 ***********************************************
 418.2210  29.7671 ******************
 419.2239   4.8696 ***
 420.2267   0.5713
 421.2295   0.0528
 422.2321   0.0040
 423.2348   0.0003
```

Figure S8. Alpha-D data and Calculated optical rotation of saccharobisindole (**1**)

Basis functions	Def-SV(P)	6-31G*	6-31G**
***R*,*R* - diastereomer**	**-798.2**	**-868.15**	**-889.46**
***R*,*S* – diastereomer**	**-12.43**	**+40.51**	**+51.93**
***S*,*R* – diastereomer**	**-725.36**	**-751.83**	**-764.03**
***S*,*S* - diastereomer**	**-252.74**	**-315.09**	**-308.47**
**Experimental optical rotation : +161**			

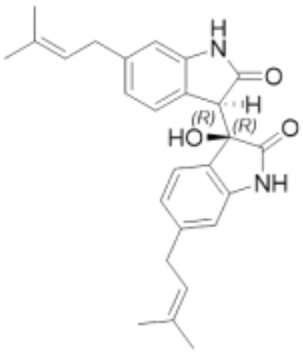

***R*,*R* - diastereomer**

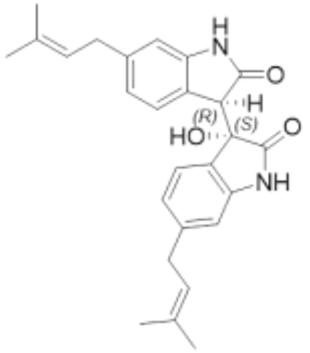

***R*,*S* - diastereomer**

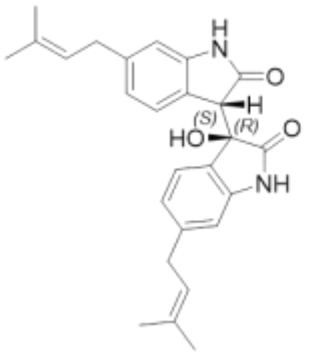

***S*,*R* - diastereomer**

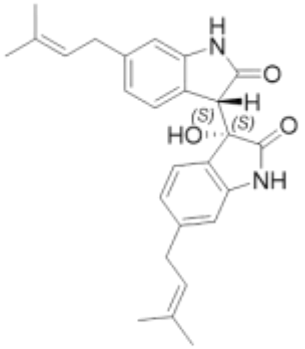

***S*,*R* - diastereomer**

Figure S9. ^{1}H NMR Spectrum (400 MHz) of neoasterric methyl ester (**2**) in DMSO-d_6

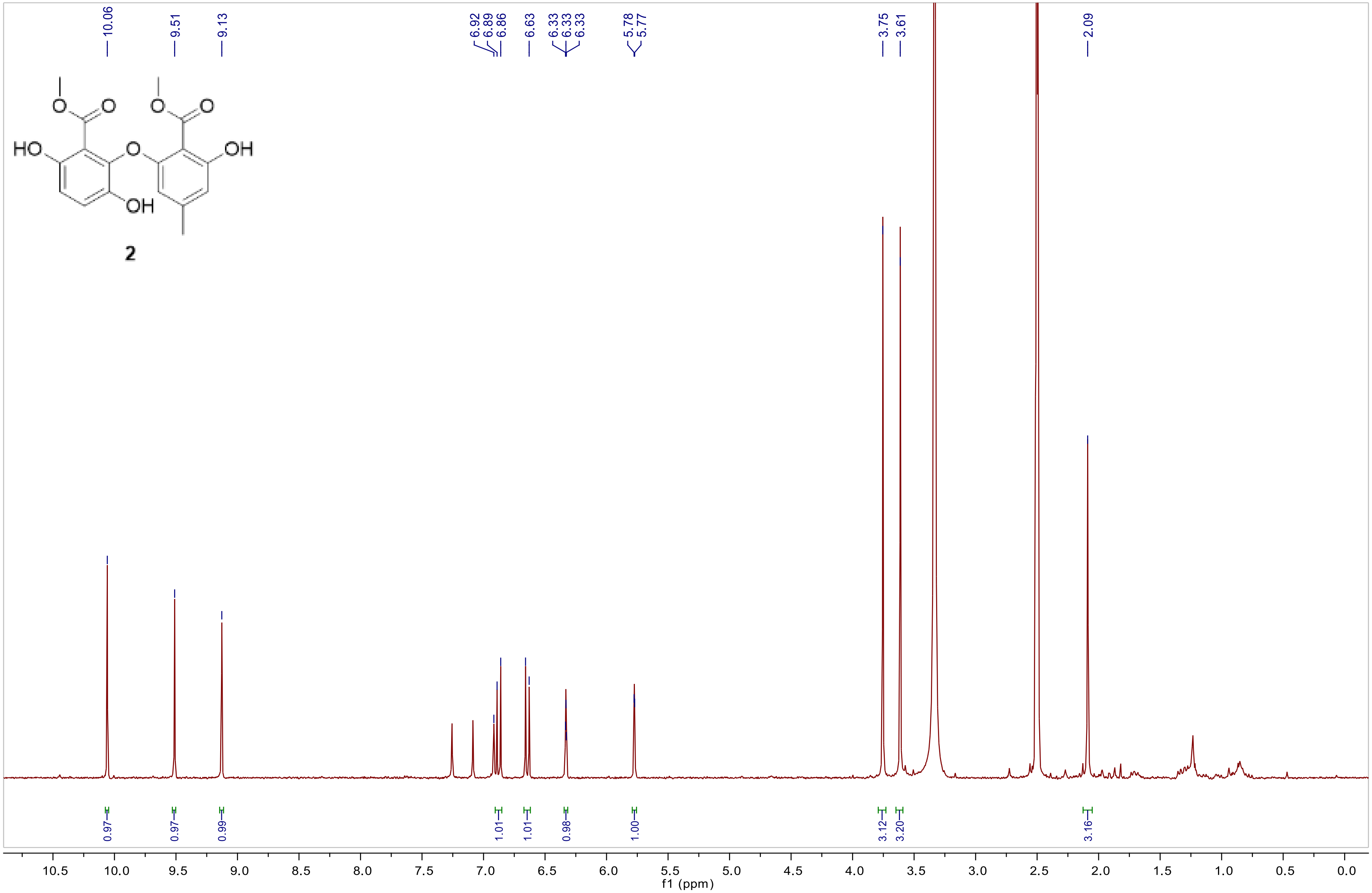

Figure S10. ^{13}C NMR Spectrum (100 MHz) of neoasterric methyl ester **(2)** in DMSO-d_6

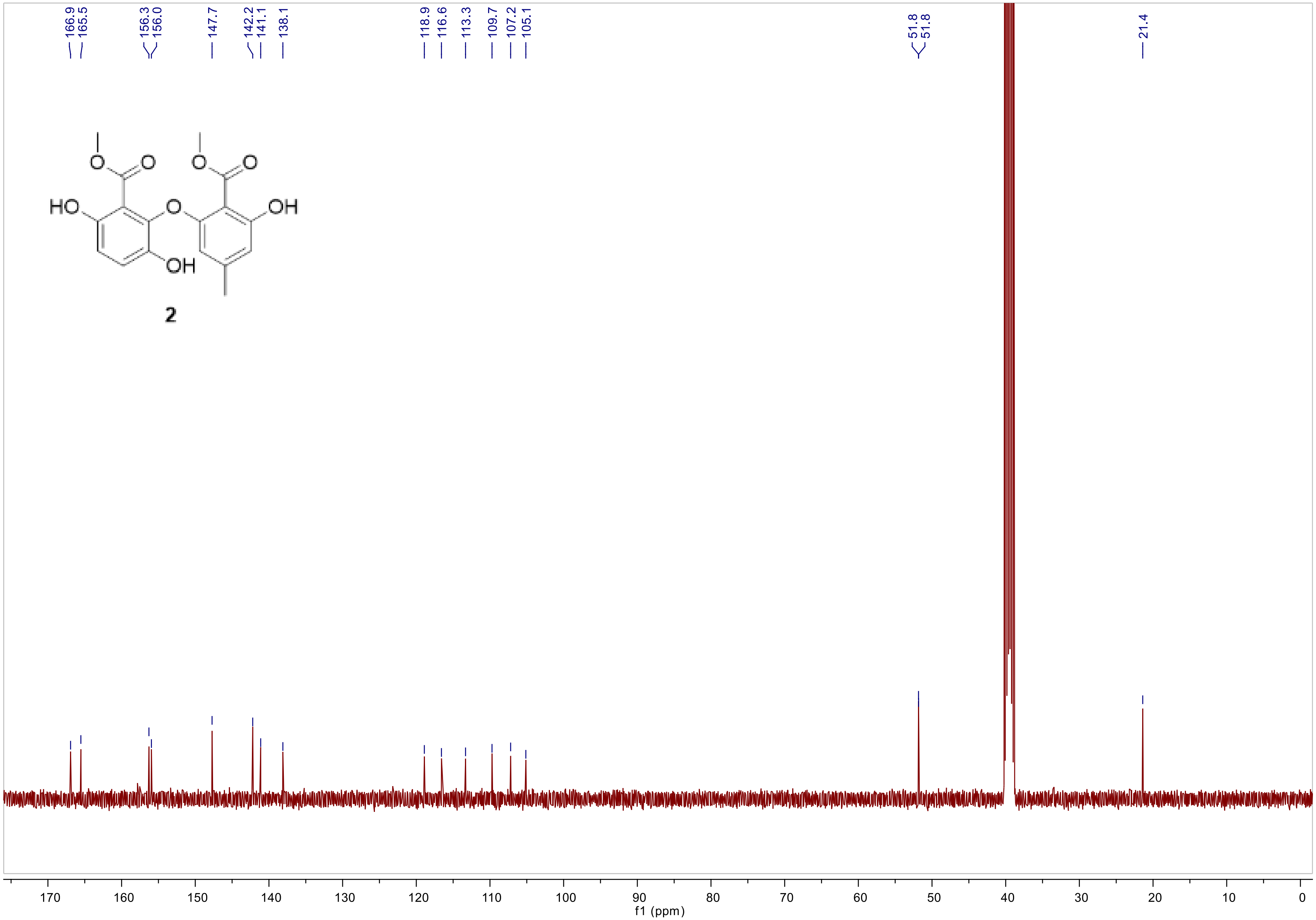

Figure S11. COSY Spectra (400 MHz) of neoasterric methyl ester (**2**) in DMSO-d_6

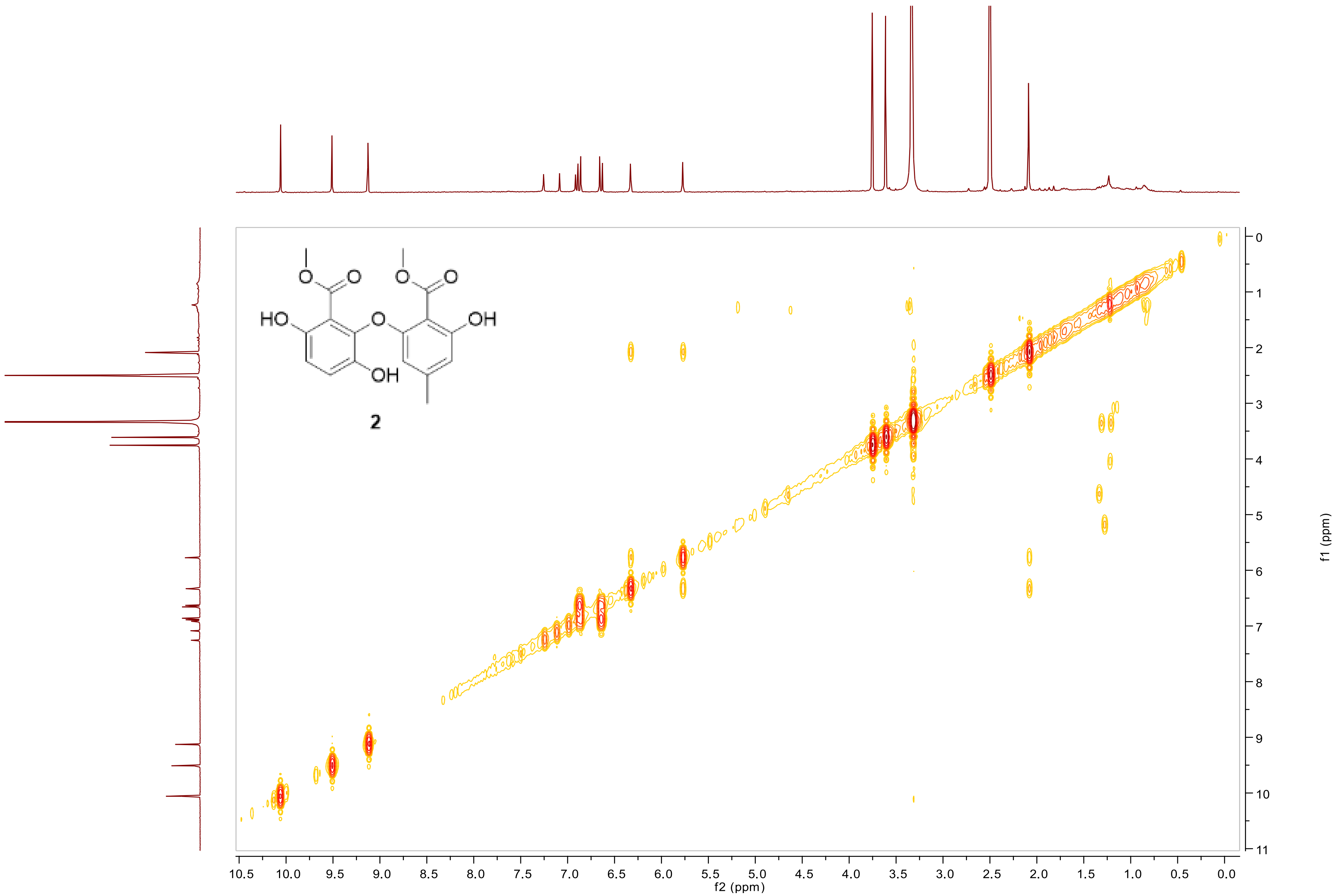

Figure S12. HSQC Spectra (400 MHz) of neoasterric methyl ester **(2)** in DMSO-d_6

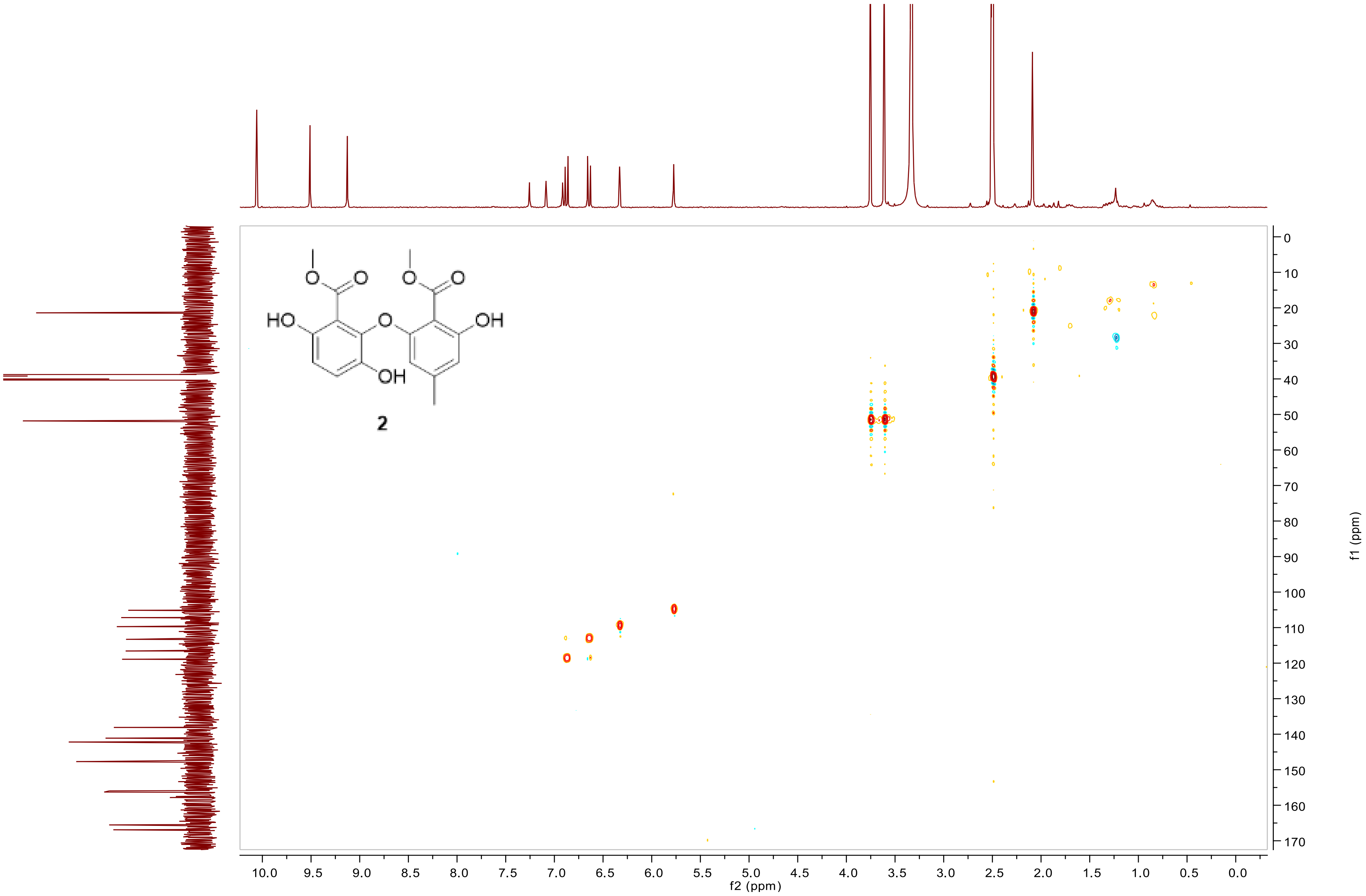

Figure S13. HMBC Spectra (400 MHz) of neoasterric methyl ester **(2)** in DMSO-d_6

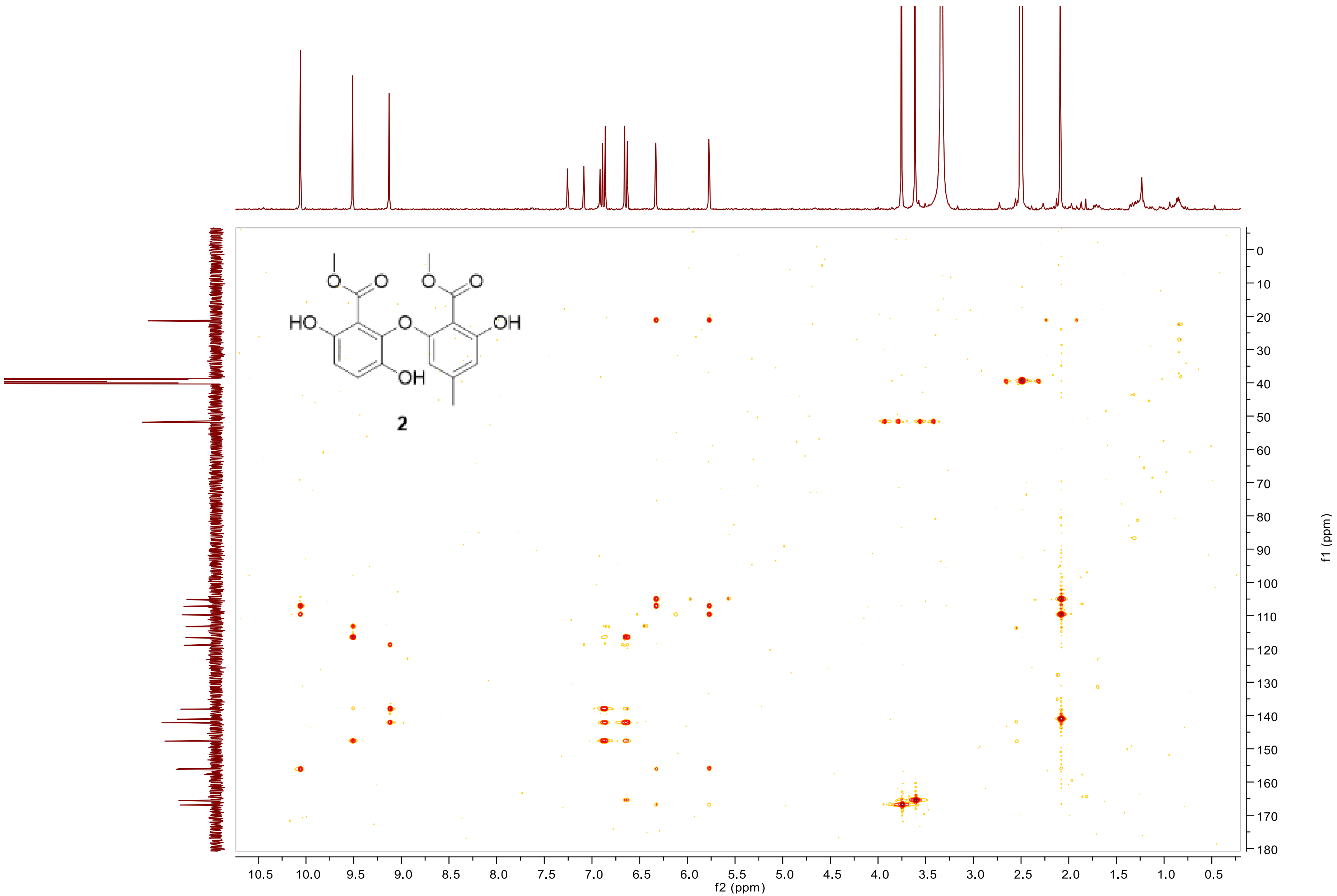

Figure S14. ^{1}H NMR spectrum (400 MHz) of 7-chloro-4(*1H*)-quinolone (**3**) in CD_3OD

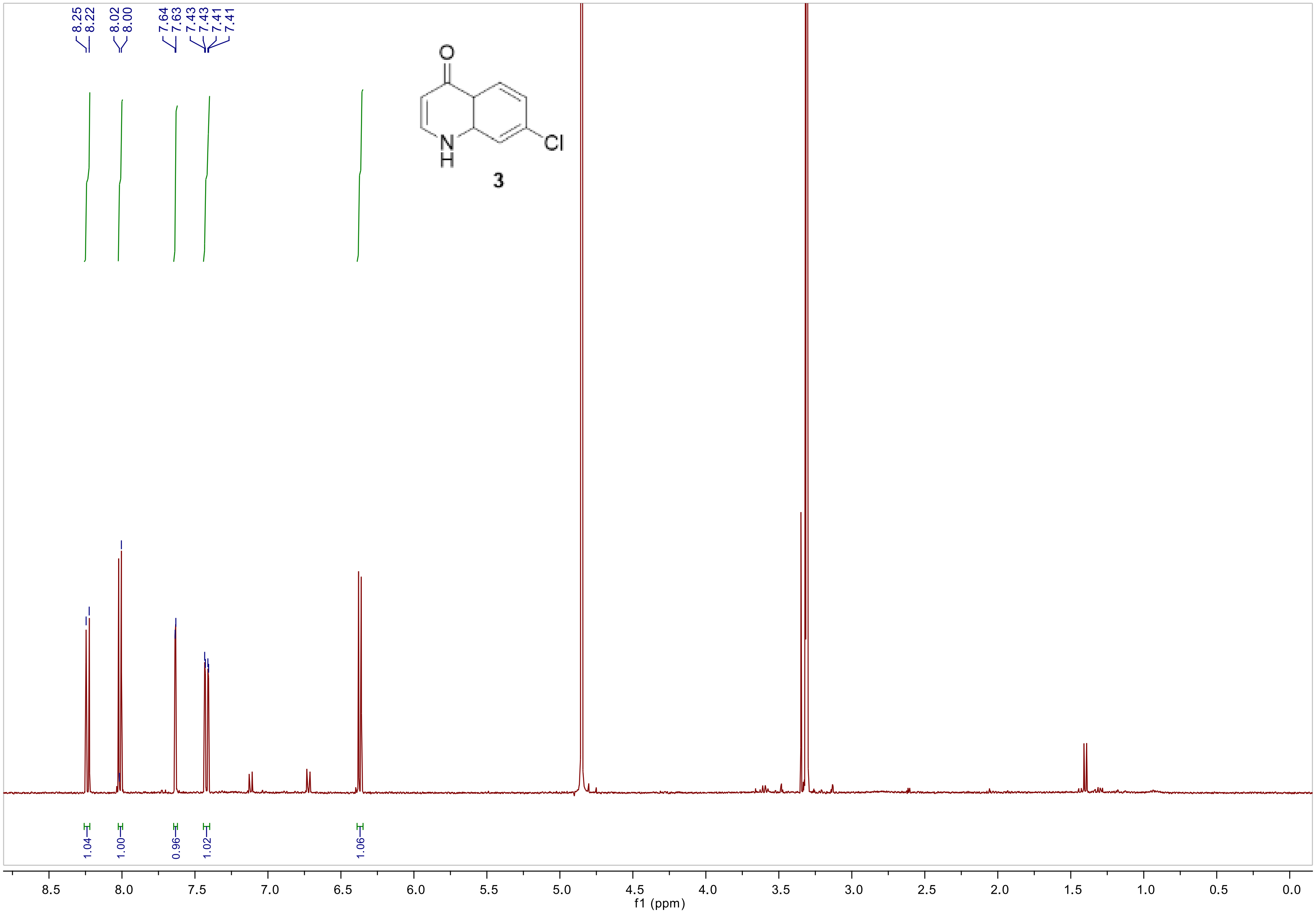

Figure S15. ^{13}C NMR spectrum (100 MHz) of 7-chloro-4(*1H*)-quinolone (**3**) in CD_3OD

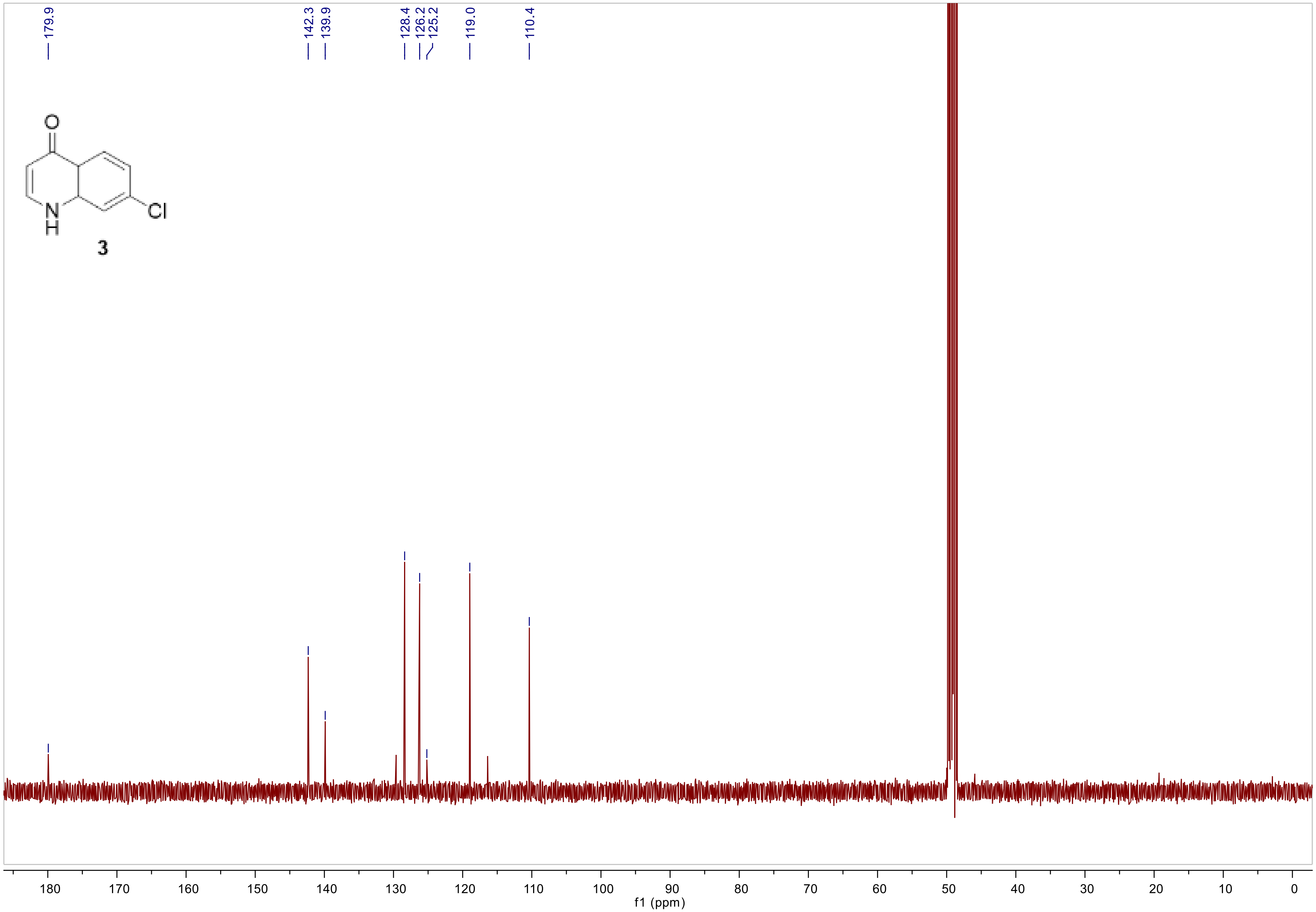

Figure S16. COSY spectrum (400 MHz) of 7-chloro-4(*1H*)-quinolone (**3**) in CD_3OD

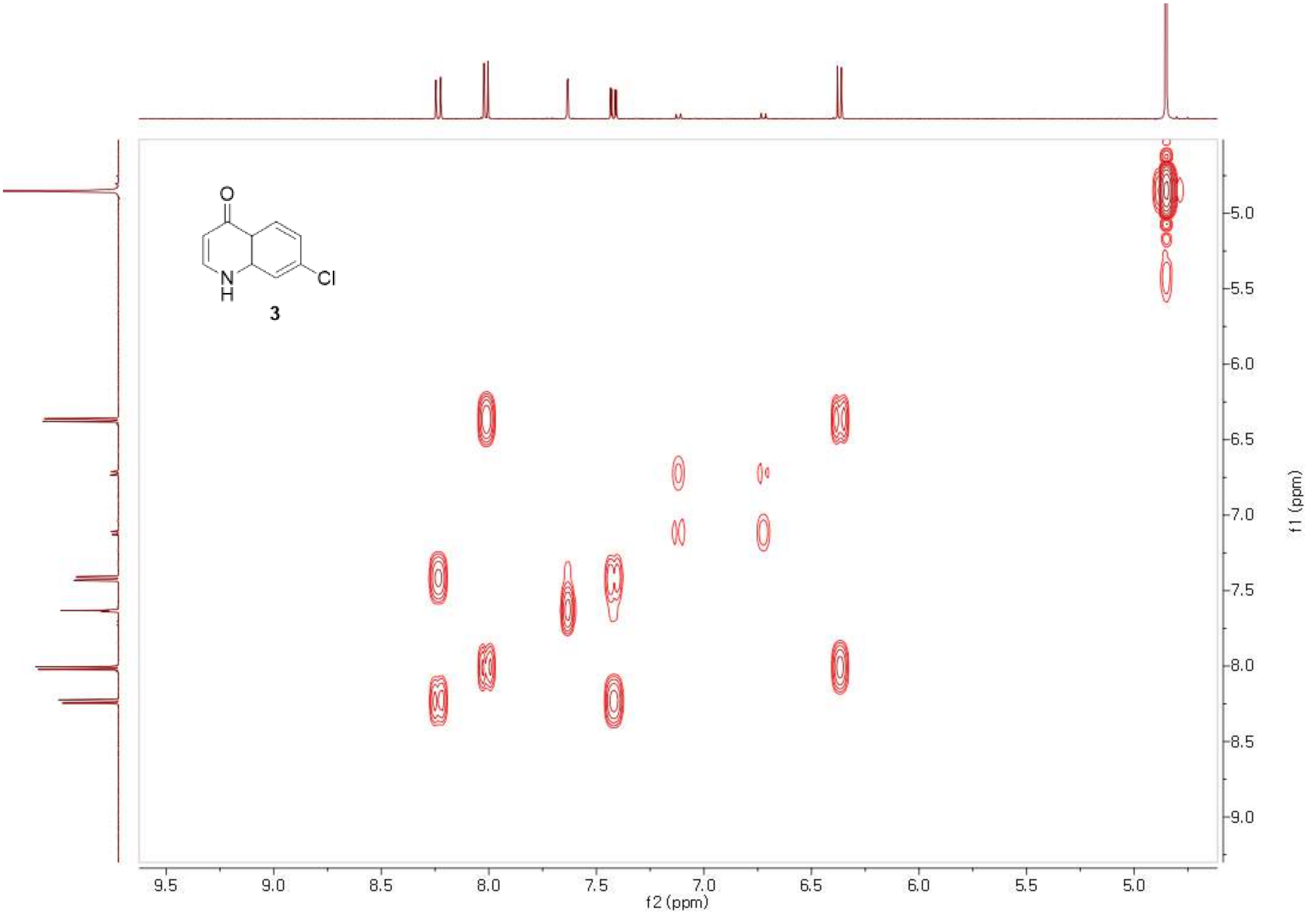

Figure S17. HSQC spectrum (400 MHz) of 7-chloro-4(*1H*)-quinolone (**3**) in CD_3OD

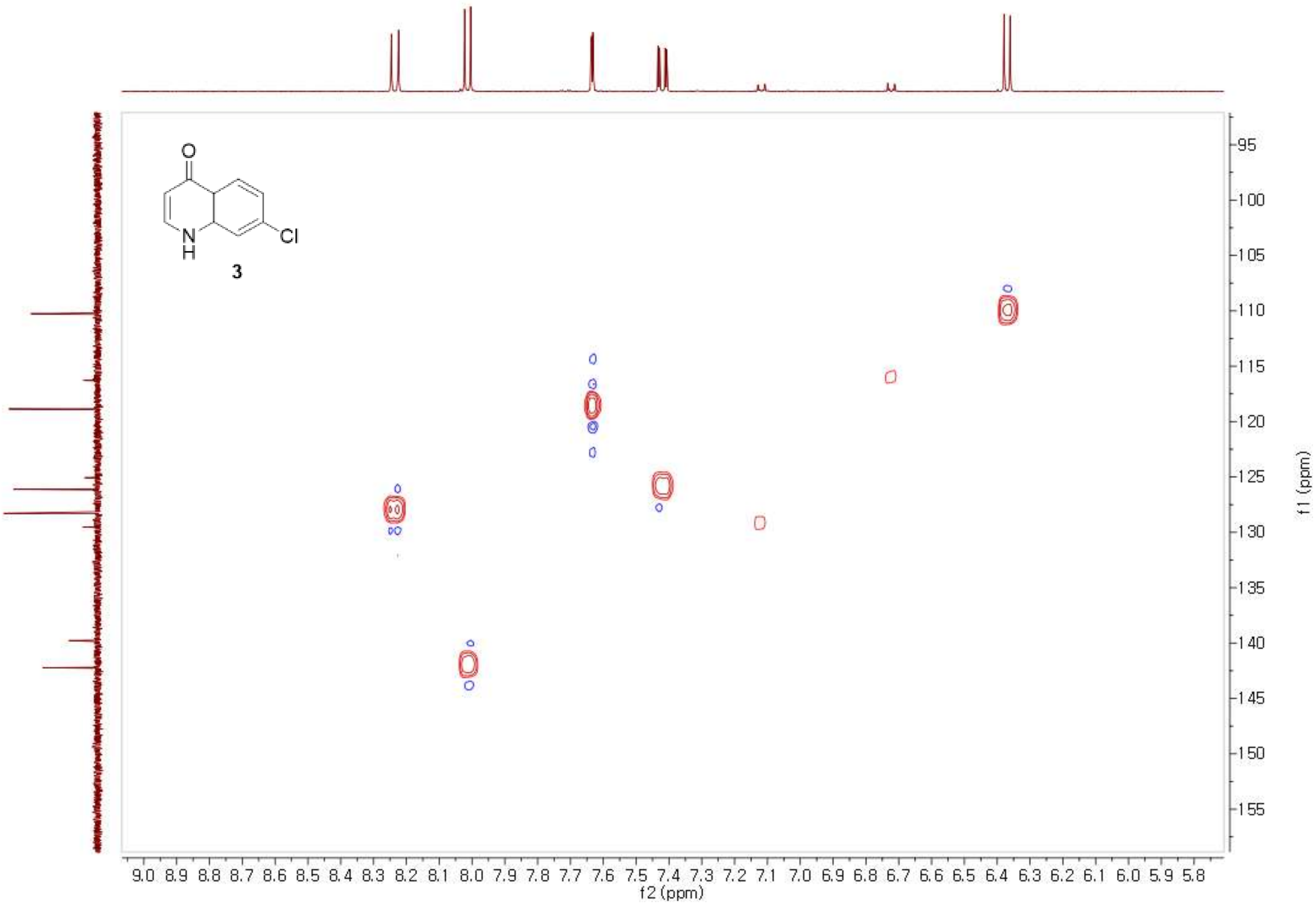

Figure S18. HMBC spectrum (400 MHz) of 7-chloro-4(*1H*)-quinolone (**3**) in CD_3OD

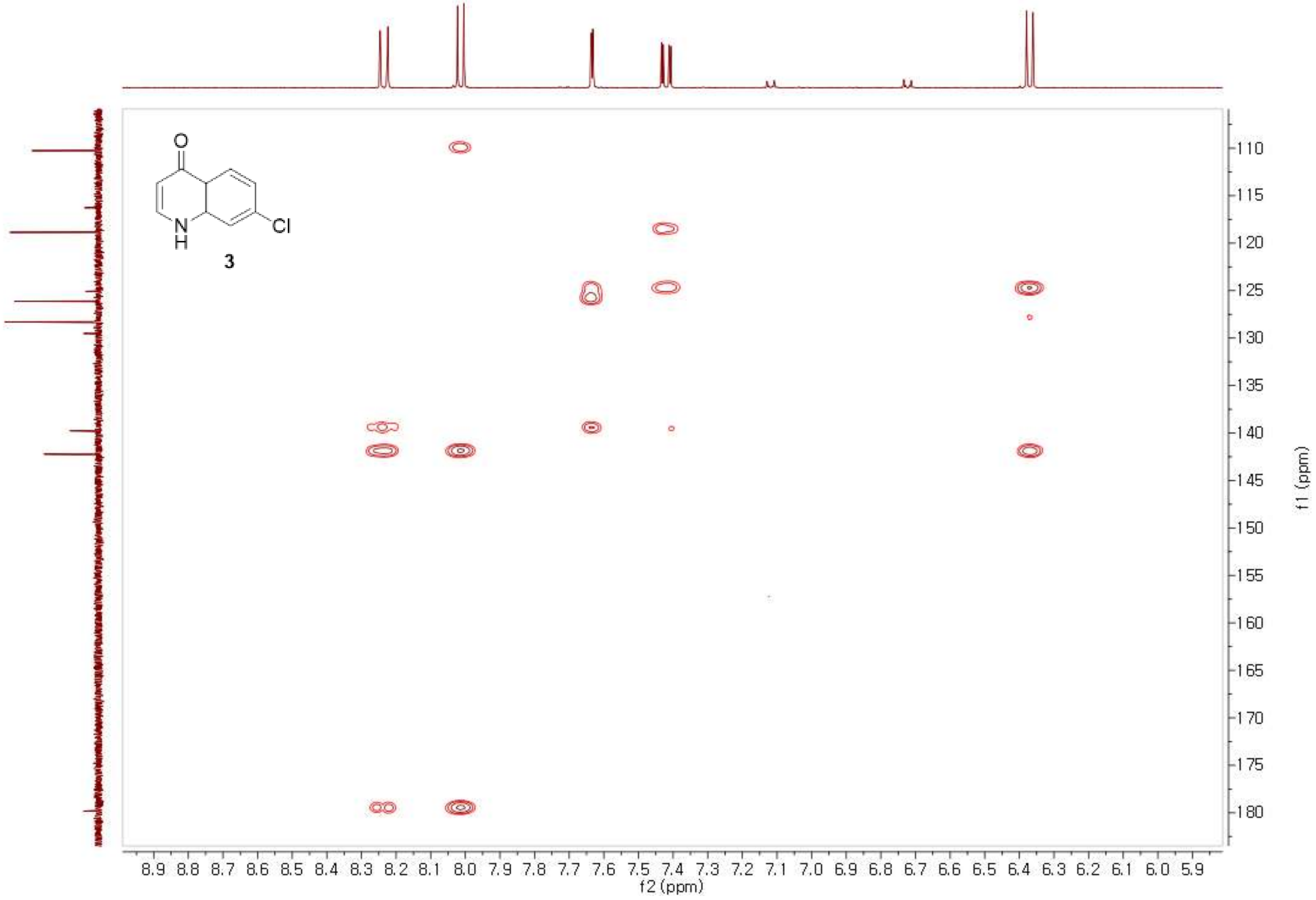

Figure S19. LR-ESI-MS spectroscopic data of 7-chloro-4(*1H*)-quinolone (**3**)

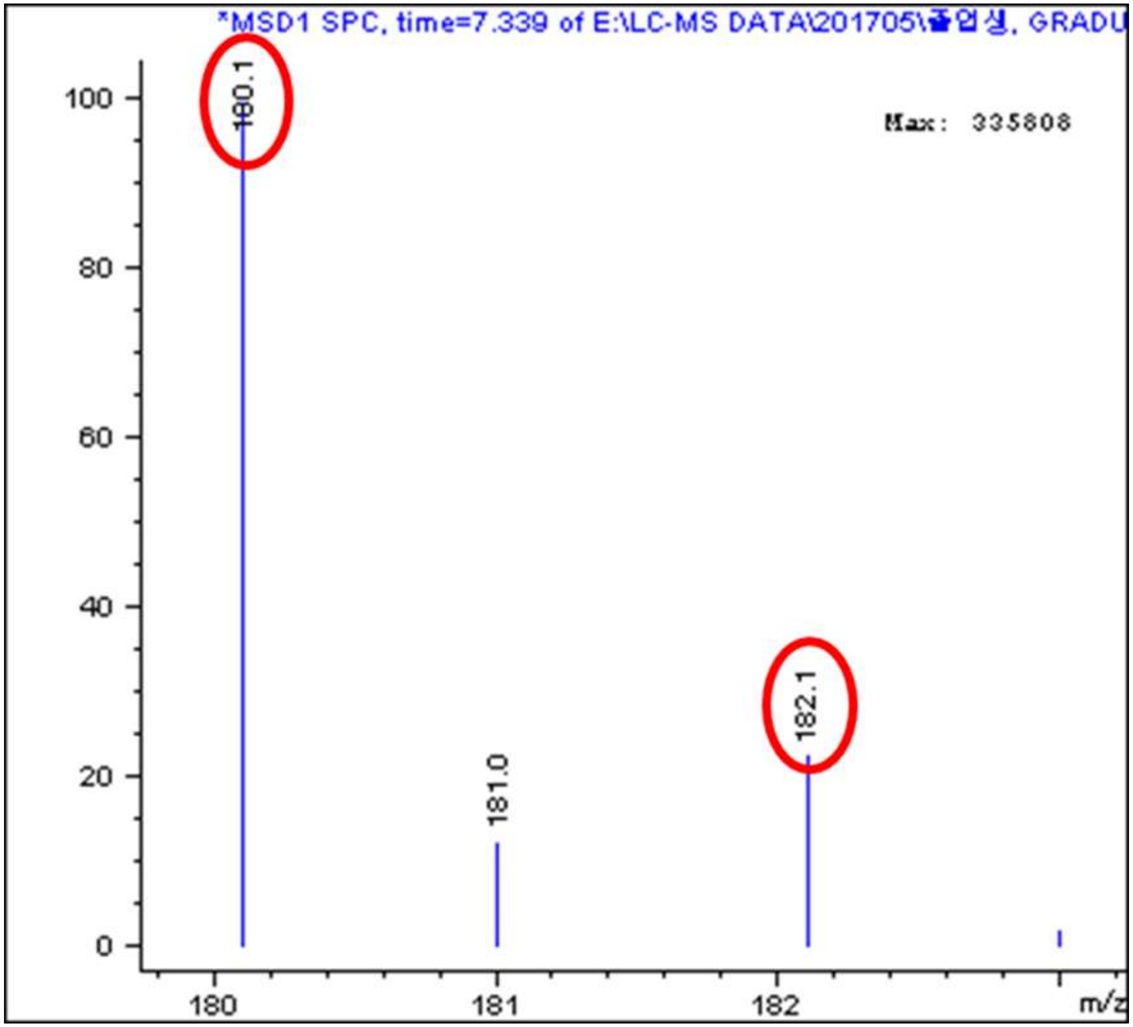

Figure S20. ^{1}H NMR Spectrum (400 MHz) of acremonidine E (**4**) in DMSO-d_6

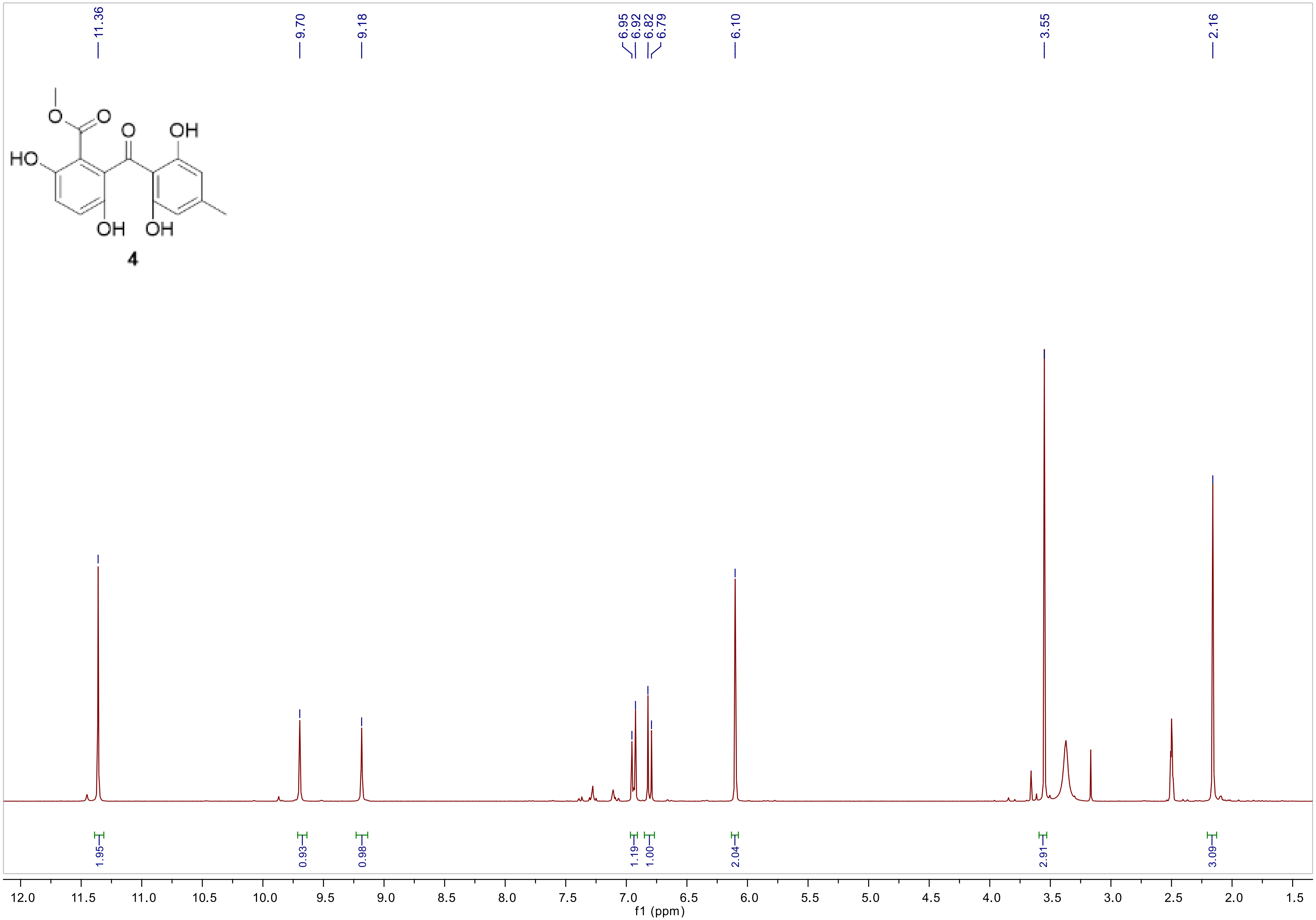

Figure S21. ^{13}C NMR Spectrum (100 MHz) of acremonidine E (**4**) in DMSO-d_6

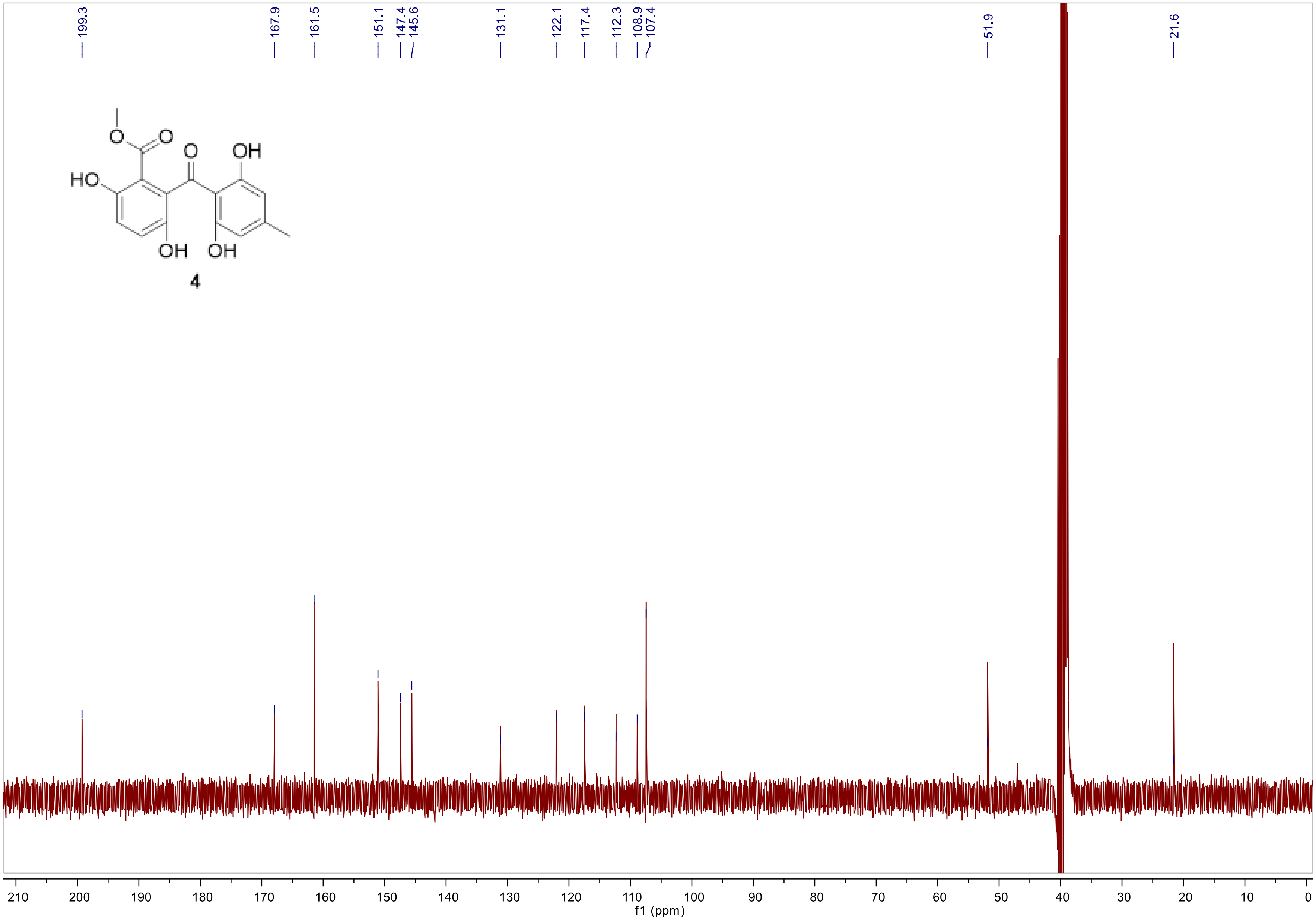

Figure S22. ^{1}H NMR Spectrum (400 MHz) of pinselin (**5**) in DMSO-d_6.

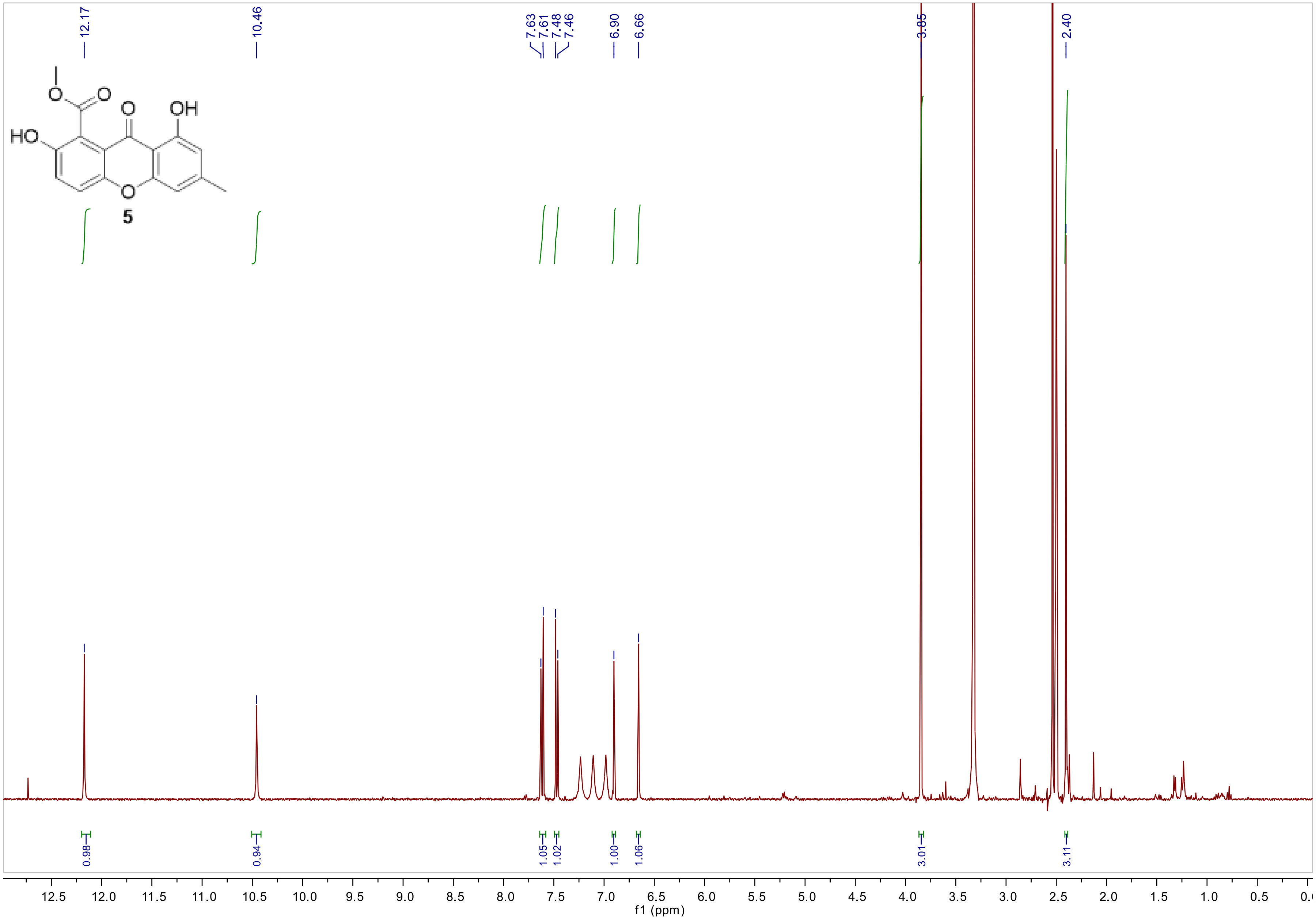

Figure S23. ^{13}C NMR Spectrum (100 MHz) of pinselin (**5**) in DMSO-d_6.

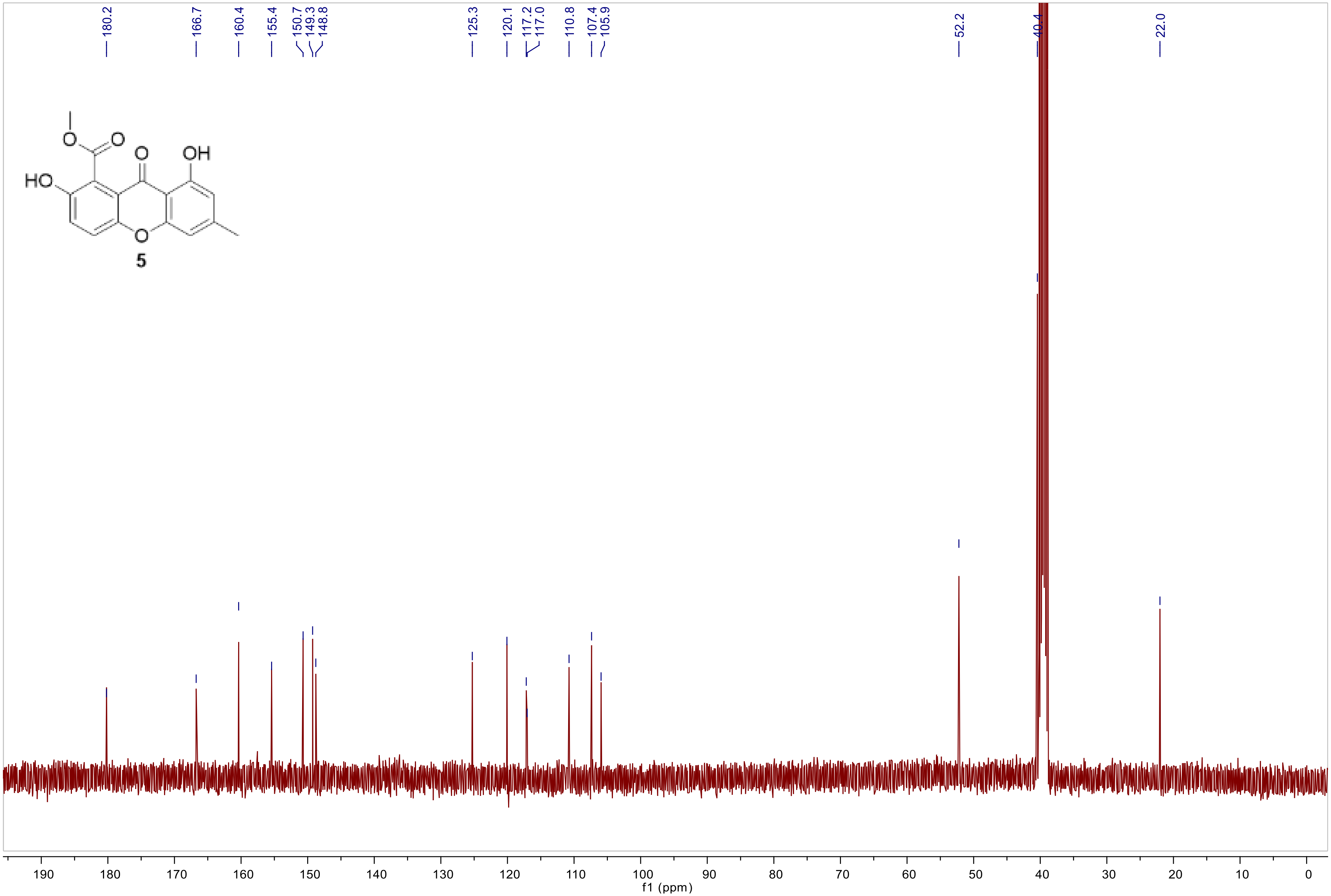

Figure S24. ^{1}H NMR Spectrum (400 MHz) of penicitirinoini A (**6**) in CD_3OD

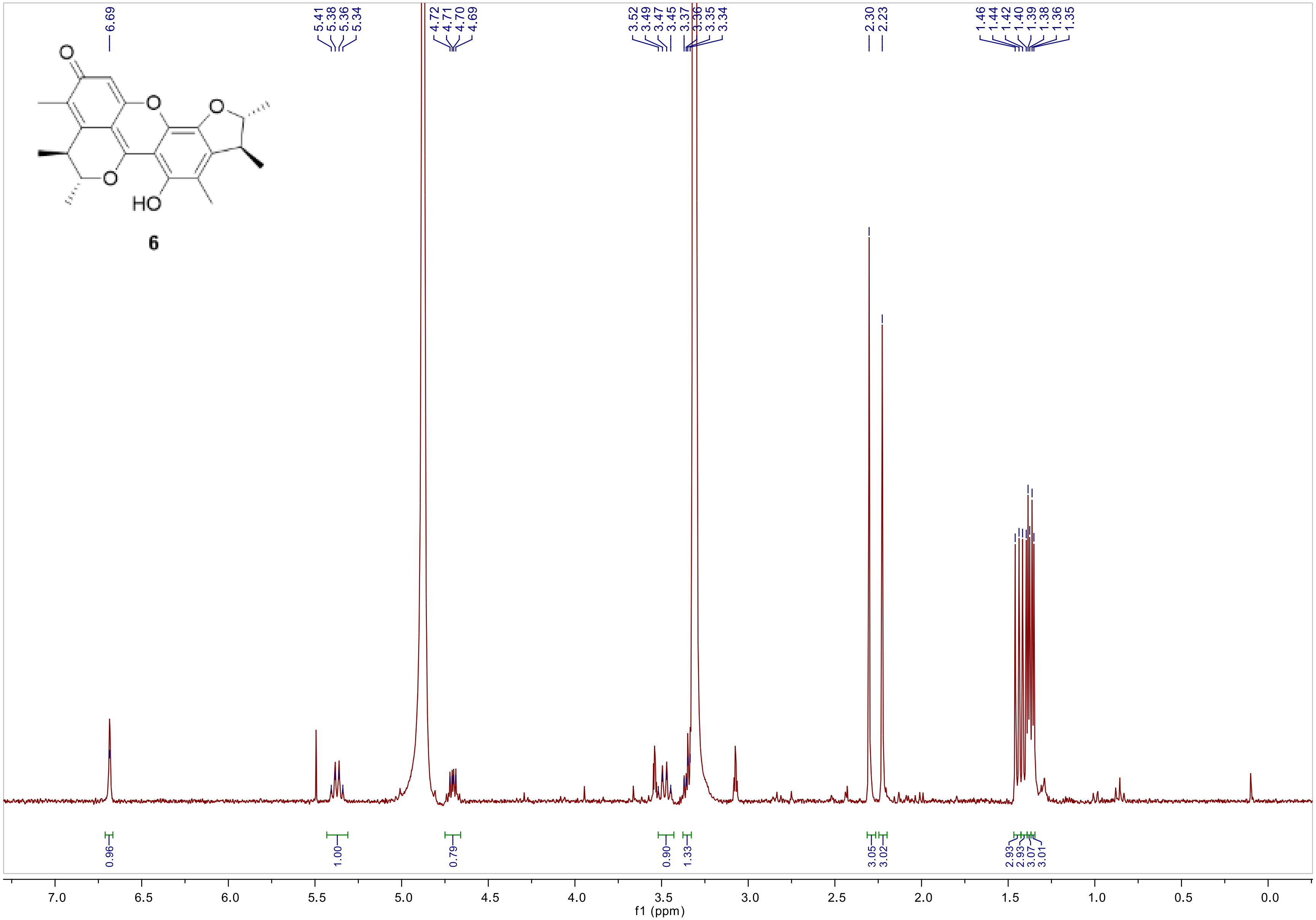

Figure S25. ^{13}C NMR Spectrum (100 MHz) of penicitirinoini A (**6**) in CD_3OD

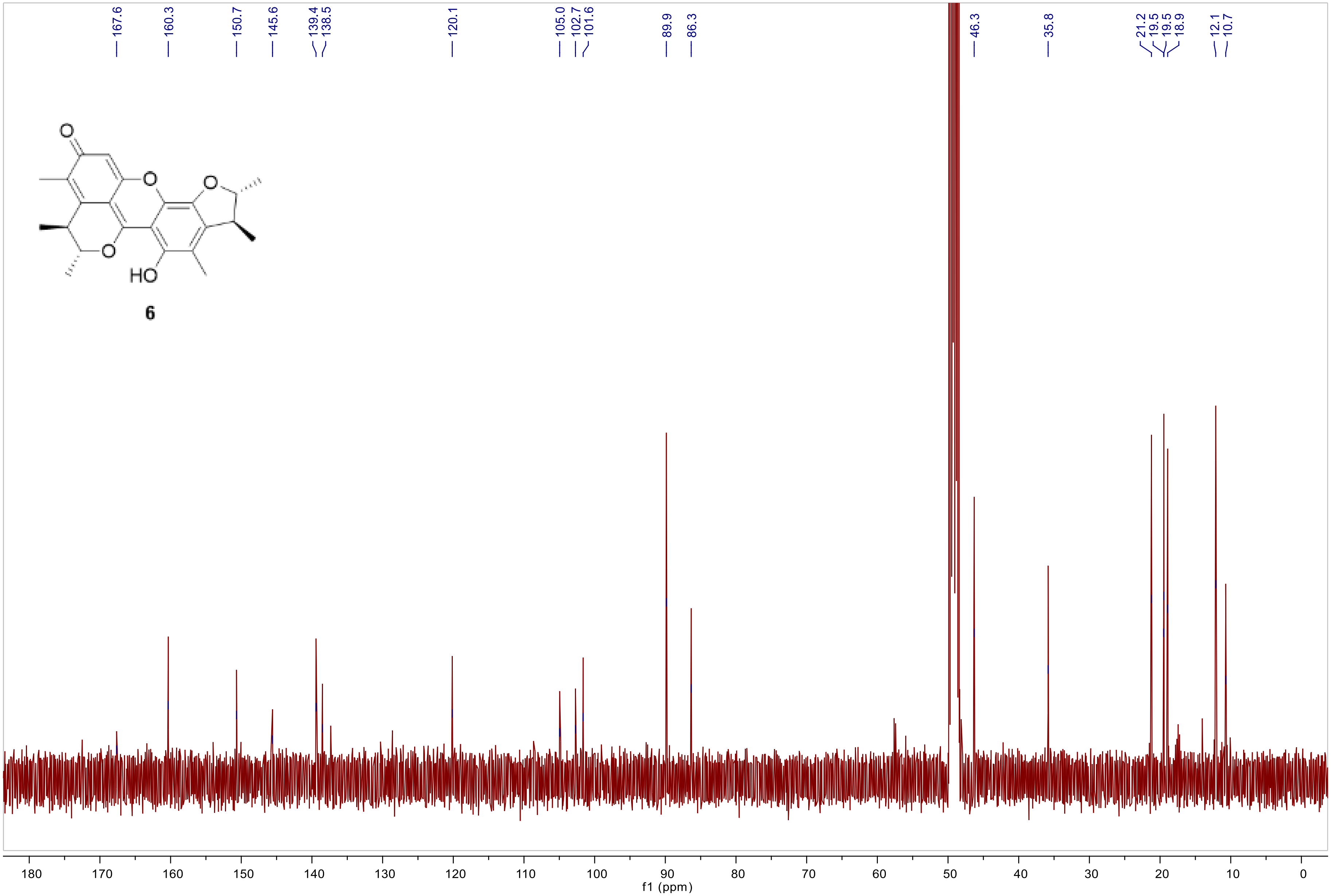

Figure S26. ^{1}H NMR Spectrum (400 MHz) of penicitirinoini E (**7**) in CD_3OD

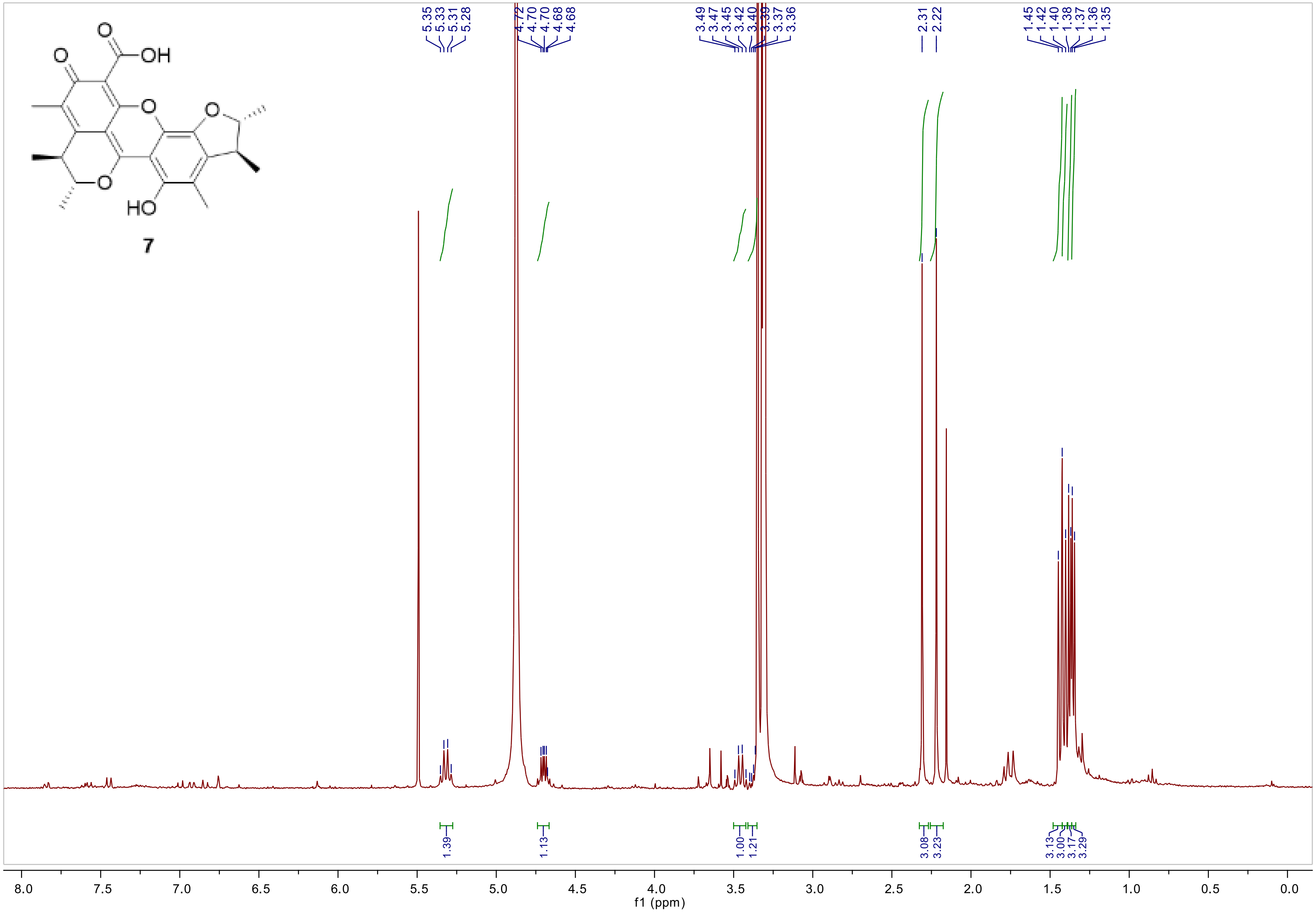

Supplement: Supplementary file 1 [file marinedrugs-20-00035-s001.zip › marinedrugs-1474927-supplementary.pdf]
